# Biostimulant Effects of *Chaetomium globosum* and *Minimedusa polyspora* Culture Filtrates on *Cichorium intybus* Plant: Growth Performance and Metabolomic Traits

**DOI:** 10.3389/fpls.2022.879076

**Published:** 2022-05-12

**Authors:** Veronica Spinelli, Elisa Brasili, Fabio Sciubba, Andrea Ceci, Ottavia Giampaoli, Alfredo Miccheli, Gabriella Pasqua, Anna Maria Persiani

**Affiliations:** ^1^Department of Environmental Biology, Sapienza University of Rome, Rome, Italy; ^2^NMR-Based Metabolomics Laboratory (NMLab), Sapienza University of Rome, Rome, Italy

**Keywords:** *Chaetomium globosum*, *Minimedusa polyspora*, fungal culture filtrates, fungi, biostimulants, plant growth promotion, *Cichorium intybus*, ^1^H-NMR based metabolomics

## Abstract

In this study, we investigated the biostimulant effect of fungal culture filtrates obtained from *Chaetomium globosum* and *Minimedusa polyspora* on growth performance and metabolomic traits of chicory (*Cichorium intybus*) plants. For the first time, we showed that *M. polyspora* culture filtrate exerts a direct plant growth-promoting effect through an increase of biomass, both in shoots and roots, and of the leaf area. Conversely, no significant effect on morphological traits and biomass yield was observed in *C. intybus* plants treated with *C. globosum* culture filtrate. Based on ^1^H-NMR metabolomics data, differential metabolites and their related metabolic pathways were highlighted. The treatment with *C. globosum* and *M. polyspora* culture filtrates stimulated a common response in *C. intybus* roots involving the synthesis of 3-OH-butyrate through the decrease in the synthesis of fatty acids and sterols, as a mechanism balancing the NADPH/NADP^+^ ratio. The fungal culture filtrates differently triggered the phenylpropanoid pathway in *C. intybus* plants: *C. globosum* culture filtrate increased phenylalanine and chicoric acid in the roots, whereas *M. polyspora* culture filtrate stimulated an increase of 4-OH-benzoate. Chicoric acid, whose biosynthetic pathway in the chicory plant is putative and still not well known, is a very promising natural compound playing an important role in plant defense. On the contrary, benzoic acids serve as precursors for a wide variety of essential compounds playing crucial roles in plant fitness and defense response activation. To the best of our knowledge, this is the first study that shows the biostimulant effect of *C. globosum* and *M. polyspora* culture filtrates on *C. intybus* growth and metabolome, increasing the knowledge on fungal bioresources for the development of biostimulants.

## Introduction

Modern agriculture is currently facing the major challenge of adapting to a rapidly evolving climate change while searching for new strategies to increase food production. Indeed, by 2050, the world population is expected to grow reaching nearly 9.7 billion requiring an increase in food production by up to 60%, while due to climate change plants already are more frequently subjected to severe abiotic and biotic stresses (Velásquez et al., 2018; United Nations Department of Economic Social Affairs Population Division, 2019; Nephali et al., 2020; Sangiorgio et al., 2020; Tian et al., 2021). This challenge is also concomitant with the need to reduce agriculture's impact on the environment, since more than 33% of soils worldwide are already degraded (Abhilash, 2021). In fact, the overapplication of agrochemicals, on which agriculture heavily relied to meet the food demand, impaired the environment, determining phenomena such as eutrophication, ecosystem simplification, loss of ecosystem services, and loss of biodiversity and of soil quality (Tilman et al., 2002; Spinelli et al., 2021; Tian et al., 2021).

Therefore, it is necessary to promote a more sustainable and resilient agriculture, based on environmentally friendly strategies and solutions, capable of preserving and restoring ecosystems and natural resources, as also affirmed in the UN 2030 Agenda Sustainable Development Goals (SDG 2.4) (United Nations, 2015). In this context, biostimulants represent an interesting sustainable solution that may play a key role in increasing crop resilience and productivity in adverse environmental conditions, minimizing agrochemicals applications and tackling climate change effects (Castiglione et al., 2021; Del Buono, 2021; Ganugi et al., 2021). Moreover, microbial biostimulants present advantageous characteristics such as not accumulating in the long term, lower toxicity, and a scarce tendency to select resistant strains of pests and pathogens compared with agrochemicals (Sangiorgio et al., 2020).

The acknowledgment of the significant role of biostimulants in the picture of a more sustainable agriculture is also reflected by their introduction as fertilizing products in the EU regulation 2019/1009 (EU, 2019). According to the new regulation, a plant biostimulant “shall be an EU fertilizing product the function of which is to stimulate plant nutrition processes independently of the product's nutrient content with the sole aim of improving one or more of the following characteristics of the plant or the plant rhizosphere: (a) nutrient use efficiency, (b) tolerance to abiotic stress, (c) quality traits, or (d) availability of confined nutrients in the soil or rhizosphere”. Concerning microbial biostimulants, the new legislation considers *in vivo*, dead or empty-cell microorganisms, and non-harmful residual elements of the media on which they were produced, but unfortunately lists only *Azospirillum* spp., *Azotobacter* spp., *Rhizobium* spp., and mycorrhizal fungi as suitable microorganisms.

Despite still not being included in the current legislation, several non-mycorrhizal fungal strains have already been reported in scientific literature as effective plant growth-promoting fungi (PGPF), and some of these have also been employed in the formulation of commercialized products (Hyde et al., 2019). Furthermore, the new legislation, to avoid regulatory conflicts with phytochemicals, does not consider the bioprotection effects associated with some biostimulants. In fact, biostimulants may also provide protection from biotic stresses, both eliciting the production of secondary metabolites inducing systemic resistance, and exerting a direct activity against pests and/or pathogens (Sangiorgio et al., 2020; Ganugi et al., 2021).

Since PGPF exert their action also through diffusible substances such as phytohormones, enzymes, amino acids, and siderophores, research focused also on the application of culture filtrates as biostimulants. Culture filtrates have been consistently reported in several studies to be effective in promoting plant growth by enhancing seed germination, biomass production, and metabolites production (Varma et al., 1999; Singh et al., 2003; Khan et al., 2008, 2015; Hamayun et al., 2009, 2010; Bagde et al., 2011, 2013; Hwang et al., 2011; Sung et al., 2011; Rahman et al., 2012; You, 2012; Murali and Amruthesh, 2015; Bilal et al., 2018; Baroja-Fernández et al., 2021; Khalmuratova et al., 2021).

*Chaetomium globosum* Kunze is a filamentous species belonging to the Ascomycota phylum. *C. globosum* presents a cosmopolitan distribution and has been reported as both a saprotroph and a plant endophyte with a wide range of host plants (Větrovský et al., 2020; Linkies et al., 2021). It presents great adaptability by inhabiting various environments including extreme habitats, such as deserts or salt lakes, and is commonly isolated in agricultural soils (Abdel-Azeem, 2020; Větrovský et al., 2020; Linkies et al., 2021). *C. globosum* is a widely studied species, and several studies highlighted the production of a wide array of bioactive metabolites such as hydrocarbons, phenols, terpenoids, and sulfur compounds, including 4-methyl-(1,5-dimethyl-4-hexenyl)-benzene, tetradecane, dodecane, hexadecane, β-bisabolene, and dimethyl-propyl-disulfide that were identified as major components, and chlorinated azaphilone derivatives such as chaetomugilins and chaetoglobosins with antifungal activity (Qin et al., 2009; Abdel-Azeem, 2020; Kumar et al., 2020, 2021). Moreover, it has already been reported as a plant growth promoter species and as an effective biocontrol agent against a large number of fungal pathogens and nematodes (Aggarwal et al., 2004, 2016; Tarafdar and Gharu, 2006; Abou Alhamed and Shebany, 2012; Biswas et al., 2012; Khan et al., 2012, 2019; Hu et al., 2013; Yan et al., 2018; Abdel-Azeem, 2020; Moya et al., 2020; Kumar et al., 2021; Linkies et al., 2021; Singh et al., 2021).

Definitely less studied is *Minimedusa polyspora* (Hotson) Weresub & P.M. Le Clair, a filamentous anamorphic species belonging to the Basidiomycota phylum. *M. polyspora* has been widely isolated in agricultural soils worldwide and found to be overrepresented in the tilled soil (Klaubauf et al., 2010; Panelli et al., 2017; Lucadamo, 2018; Longley et al., 2020; Větrovský et al., 2020; Orrù et al., 2021). This species presents several traits such as rapid growth, low requirement of nitrogen, ability to concentrate important biogenic macroelements (N, P, S, K, and Ca) and translocate nutrients, metabolic plasticity, and the production of antibiotic and antifungal compounds (triene compounds), which make it an efficient pioneer colonizer (Beale and Pitt, 1995; Pinzari et al., 2014). Furthermore, *M. polyspora* has already been suggested to be a PGPF given its ability to solubilize inorganic phosphorous and to antagonize *Fusarium oxysporum* f. sp. *narcissi* (Beale and Pitt, 1990, 1995; Ceci et al., 2018); nevertheless, to the best of our knowledge, no further studies with a wider focus than *Fusarium* biocontrol have yet been reported on its direct effect on plant growth promotion.

*Cichorium intybus* L., commonly known as chicory, is a perennial herb belonging to the *Asteraceae* family. Native to Europe, Northern Africa, and Mid-Asia, *C. intybus* has been introduced also in Northern America, Southern America, India, Asia, Australia, and New Zealand reaching nowadays a cosmopolitan distribution (Wang and Cui, 2011; Al-Snafi, 2016; Kew Science, 2021).

Chicory, cultivated since the III century before Christ for several purposes, has also been historically reported as a medicinal plant commonly utilized in the traditional medicine of several countries in Europe, Africa, and Asia (Wang and Cui, 2011; Street et al., 2013; Puhlmann and de Vos, 2020). *C. intybus* extracts have been reported in recent years to possess analgesic, anti-inflammatory, antimalarial, antimicrobial, anticancer, anti-neurotoxic, antiviral, hypotensive, hepatoprotective, and anti-protozoal and antiparasitic properties (Bischoff et al., 2004; Street et al., 2013; Al-Snafi, 2016; Janda et al., 2021).

These multiple functions of chicory are deeply related to its rich and complex phytochemical profile including a great number of bioactive substances. Despite being more abundant in the roots, important phytochemicals are reported to be distributed throughout the whole plant including sesquiterpene lactones, caffeic acid derivatives, organic acids, inulin, flavonoids, polyphenols, alkaloids, steroids, fats, proteins, hydroxycoumarins, terpenoids, oils, glycosides, volatile compounds, vitamins, β-carotene, zeaxanthin, β-sitosterol, tannins, and minerals (Bais and Ravishankar, 2001; Street et al., 2013; Puhlmann and de Vos, 2020; Janda et al., 2021; Perović et al., 2021). This rich profile presents multiple nutritionally important compounds, including among the most important ones: carbohydrates, phenolic compounds, flavonoids, amino acids and proteins, fatty acids, sesquiterpene lactones, vitamins, minerals, and micronutrients (Perović et al., 2021). Thanks to its valuable nutritional values and its health-promoting characteristics, chicory is commonly used as a vegetable, coffee substitute, forage, and functional ingredient in commercial food products (Puhlmann and de Vos, 2020; Janda et al., 2021; Perović et al., 2021). A wide range of chicory cultivars, selected on the basis of their suitability for each purpose (e.g., leafy salad, root production, and forage), are currently available (Wang and Cui, 2011).

In this study, we aimed at evaluating the effect of the application of fungal culture filtrates of *M. polyspora* and *C. globosum*, as biostimulants, on the growth and metabolism of *C. intybus* plants. Our study has been structured to address: a) the evaluation of the effectiveness of culture filtrates in promoting plant growth, through the assessment of morphological and physiological parameters of growth; b) the evaluation of metabolism modification after culture filtrate application, addressing possible mechanisms of action of biostimulation. To the best of our knowledge, these fungal species have not been previously studied to promote *C. intybus* growth, and we, therefore, aimed at increasing the knowledge on fungal bioresources for the development of biostimulants applicable for a sustainable cultivation of this species that is of agronomic and medicinal interest.

## Materials and Methods

### Fungal Strains and Culture Filtrates Production

Two fungal strains, isolated in previous studies and currently preserved at the culture collection of the Fungal Biodiversity Laboratory (FBL) (Sapienza, University of Rome), *C. globosum* Kunze FBL 205 and *M. polyspora* (Hotson) Weresub & P. M. LeClair FBL 503, were studied to assess the ability of their culture filtrate to promote plant growth. Prior to the experiment, the strains were reactivated and maintained on malt extract agar (MEA) at 25°C in the dark. MEA was prepared according to the following composition (g/L in distilled water): malt extract, 20; peptone, 1; dextrose, 20; and bacto agar, 20. All components were purchased from Becton Dickinson (Sparks, MD, USA). Each strain's culture filtrate was prepared by inoculating four 4-mm diameter plugs of mycelium, taken from the actively growing margin of a 10-day-old stock culture using a sterile cork borer, in 150 mL Erlenmeyer flask containing 60 mL of malt extract broth (MEB). MEB was prepared with the same abovementioned composition of MEA excluding bacto agar. Five replicates were set up for each strain, and 5 Erlenmeyer flasks were left uninoculated for the control treatment. The flasks were incubated at 25°C on an orbital shaker (ASAL 711/D) at 100 rpm for 14 days.

At the end of the incubation period, the culture medium was recovered and filtered using sterile syringe filters with a 0.45-μm pore size made of mixed cellulose esters (ClearLine^®^, Dominique Dutscher SAS, Brumath, France). Culture filtrates from different biological replicates of the same fungal strain were pooled together, and a sample was recovered to perform ^1^H-NMR-based metabolomics analysis to identify the metabolites released by the fungus.

### Plant Material

Wild chicory (*C. intybus*) seeds (Fratelli Ingegnoli Spa, Milano, Italy) were surface sterilized for 20 min in a 20% ethanol solution of 15% sodium hypochlorite, followed by 5 rinses in sterile distilled water. Sterilized seeds were plated on Murashige and Skoog (MS) medium (Duchefa Biochemie, Haarlem, The Netherlands) supplemented with 30 g/L of sucrose (Carlo Erba) and 5 g/L of bacto agar (Becton Dickinson Sparks, MD, USA). Ten chicory seeds were placed in each petri dish. Petri dishes were incubated at 25°C under a photoperiod of 16/8 h (light/dark) to promote seed germination. After 15 days, seedlings were transferred to disposable plastic pots of diameter 13 cm and height 10 cm, containing approximately 265 g of autoclaved universal potting soil (COMPO SANA^®^ Universal potting soil, COMPO Italia Srl, Cesano Maderno, Italy). Pots were incubated in a walk-in chamber at 18/22°C under a photoperiod of 15/9 h (light/dark) and watered every 3 days.

### Application of the Fungal Culture Filtrates

**Figure 1:**
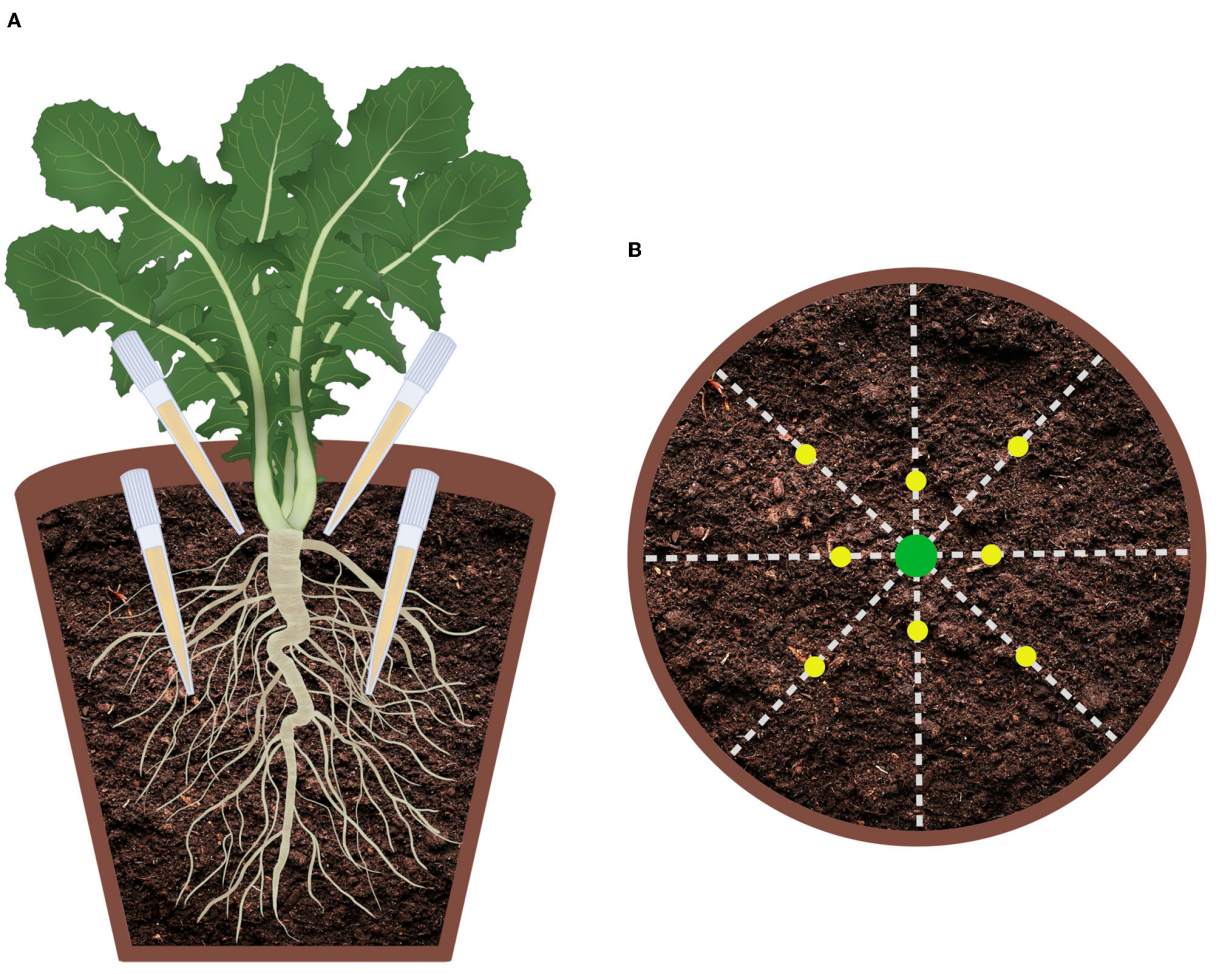
Graphical representation of culture filtrate application design. **(A)** Virtual vertical section of the pot; **(B)** Top view of the pot.

One month after the seedlings were transferred into the pots, 8 mL of the culture filtrate was directly added to the soil of each pot (concentration 30 mL/kg) in 8 different points positioned at two depths according to the scheme reported in Figure 1. In addition to the two treatments with *M. polyspora* (503) and with *C. globosum* (205) culture filtrates, two control groups treated with a corresponding volume of distilled water (control water) or with a corresponding volume of uninoculated MEB (MEB) were set up. On the treatment day, plants were watered in the morning and let draining excess for 5/6 h prior to the culture filtrate inoculation. Ten plants were randomly picked and assigned to each treatment.

### Assessment of Growth of *Cichorium intybus* Plants

Fourteen days after the culture filtrate addition, several parameters were measured to assess the plant growth. Growth parameters of five replicates randomly picked for each treatment were evaluated. Plants were removed from the pots and carefully washed to remove soil assuring to avoid root biomass loss. The root biomass was separated into taproot and secondary roots and then dried at 70°C for 48 h in the oven and weighted. The number of leaves per plant was recorded. The leaves were cut at the base, wrapped in a moist paper towel, and stored in containers overnight at 4°C. To determine the total leaf area per pot, water-saturated leaves were blotting dried and their images were acquired and later analyzed using the software ImageJ (version 1.53c, Wayne Rasband, National Institutes of Health, Bethesda, USA. Available online: https://imagej.nih.gov/ij). Following the image acquisition, shoot biomass was oven-dried at 70°C for 48 h to determine the dry weight. Mean specific leaf area (SLA) per pot (total leaf area divided by total dry weight) and root/shoot ratio were also calculated.

### Sample Preparation, ^1^H-NMR Spectra Acquisition, and Processing

Fourteen days after the culture filtrate addition, five replicates randomly picked were recovered for untargeted metabolomic analysis by ^1^H-NMR for each of the treatments: MEB control (Ctrl), *C. globosum* (205), and *M. polyspora* (503). Plants were removed from the pots, washed from the soil, and separated into the root and shoot biomass. Both the fractions were immediately frozen with liquid nitrogen to ensure the metabolic quenching and stored at −80°C. For both leaves and roots, 1.5 g of biomass grounded in liquid nitrogen was extracted according to a modified Bligh-Dyer protocol (Giampaoli et al., 2021) using a mixture of methanol, chloroform, and distilled water (3:3:1.2 mL). After overnight incubation at 4°C, samples were centrifuged at 10,000 g for 25 min at 4°C on an Itettich Zentrifugen centrifuge (Germany), and hydroalcoholic and chloroformic fractions were separated and dried under N_2_ flow. The dried phases were stored at −80°C until the NMR analysis. The dried residue of the hydro-alcoholic phase was dissolved in 0.6 mL CD_3_OD/D_2_O (1:2 v/v ratio) containing 3-(trimethylsilyl)-propionic-2,2,3,3,-d_4_ acid sodium salt (TSP, 2 mM) as internal standard (chemical shift reference). The dried residue of the chloroformic phase was dissolved in 0.6 mL CDCl_3_ (Cambridge Isotope Laboratories, Inc.) (99.8%) containing 1,1,3,3,5,5-hexamethylcyclo-tri-siloxane (HMS) (Sigma-Aldrich, USA) as internal standard (2 mM). All spectra were recorded at 298 K on a Jeol JNM-ECZ 600R spectrometer operating at the proton frequency of 600 MHz and equipped with a multinuclear z-gradient inverse probe head.

Hydroalcoholic ^1^H spectra were acquired employing the *presat* pulse sequence for solvent suppression with 128 transients, a spectral width of 9013.7 Hz, and 64 k data points for an acquisition time of 7.3 s. The recycle delay was set to 7.7 s in order to achieve complete resonance relaxation between successive scansions.

Chloroform ^1^H spectra were acquired employing a single-pulse sequence with 128 transients, a spectral width of 9013.7 Hz, and 64 k data points for an acquisition time of 7.3 s. The recycle delay was set to 7.7 s in order to achieve complete resonance relaxation between successive scansions.

Resonance assignment was carried out on the basis of 2D ^1^H-^1^H TOCSY and ^1^H-^13^C HSQC experiments. ^1^H-^1^H TOCSY spectra were acquired with 64 scans, a spectral width of 9013.7 Hz in both dimensions, a data matrix of 8 k × 256 data points, a recycle delay of 3 s, and a mixing time of 90 ms; ^1^H-^13^C HSQC spectra were acquired with 128 scans, a spectral width of 9013.7 Hz, and 30,000 Hz for hydrogen and carbon dimension, respectively, a data matrix of 8 k × 256 data points, a recycle delay of 3 s, and a direct constant of 145 Hz.

Fungal culture filtrate analysis was carried out by adding to 0.35 mL of filtrate an amount of 0.35 mL of D_2_O containing 3-(trimethylsilyl)-propionic-2,2,3,3-d_4_ acid sodium salt (TSP, 2 mM) as internal chemical shift and concentration standard, and the sample was analyzed by ^1^H-NMR employing the *presat* pulse sequence.

### Statistical Analysis

All statistical analyses on growth parameters were carried out using the statistical software R (version 4.1.0) under the R-studio environment (version 1.4.1106). For all parameters belonging to the morphological dataset, the normality and homoscedasticity of the data were tested using Shapiro-Wilk and, as appropriate, Bartlett or Levene test, respectively (packages lawstat and stats). Hereafter, differences between groups were tested using the Mann-Whitney U test, performed through the “Wilcoxon rank sum exact test” function (package stats).

Multivariate PCA and PLS-DA were performed on the data matrix using the Unscrambler ver. 10.5 software (Camo Software AS, Oslo, Norway). Univariate *t*-test and Pearson's correlation coefficients were calculated with SigmaPlot 14.0 software (Systat Software Inc., San Jose, CA, USA).

## Results

### Effects of the Application of Fungal Culture Filtrate on *Cichorium intybus* Growth

The values of growth parameters, evaluated 14 days after culture filtrate application, are reported in Table 1. Plant growth promotion effect, in terms of increase of biomass production, has been observed only in plants treated with *M. polyspora* culture filtrate. In fact, a statically significant difference (*p* < 0.05) in the total dry weight of the plant has been observed solely by comparing *M. polyspora* treatment with both water and MEB control, and the same significant result has been observed considering the dry weight of the shoot. Regarding the dry weights of the total root system and of the lateral roots, the only significant difference (*p* < 0.05) observed is between *M. polyspora* treatment and the MEB control, while no statistically significant differences among all treatments have been recorded in the taproot dry weight.

**Table 1:** Values of evaluated growth parameters.

**Parameter**	**Treatment**			
	**Water control**	**MEB control**	***C. globosum***	***M. polyspora***
Dry weight total plant (g/plant)	2.90 ± 0.12	2.96 ± 0.03	3.00 ± 0.28	3.59 ± 0.19 ^*^,^•^
Dry weight shoot (g/plant)	2.23 ± 0.04	2.38 ± 0.06	2.30 ± 0.23	2.83 ± 0.14 ^*^,^•^
Dry weight total root system (g/plant)	0.66 ± 0.10	0.58 ± 0.03	0.70 ± 0.08	0.76 ± 0.06 ^•^
Dry weight taproot (g/plant)	0.19 ± 0.03	0.18 ± 0.02	0.20 ± 0.03	0.16 ± 0.02
Dry weight lateral roots (g/plant)	0.47 ± 0.07	0.41 ± 0.04	0.50 ± 0.70	0.60 ± 0.07 ^•^
Number of leaves	28.80 ± 1.59	33.40 ± 4.01	31.60 ± 2.01	42.40 ± 5.72
Leaf area (cm^2^)	823.86 ± 31.61	789.50 ± 25.02	740.79 ± 42.83 ^▴^	996.20 ± 66.39 ^▴^,^•^
Specific leaf area index (m^2^/kg)	36.90 ± 1.17	33.35 ± 1.72	33.36 ± 3.49	35.07 ± 0.95
Root/shoot ratio	0.30 ± 0.04	0.25 ± 0.02	0.31 ± 0.03	0.27 ± 0.01

*The data are expressed as the mean ± standard error of independent replicates (n = 5)*.

* *Statistically significant compared with water control*;

• *Statistically significant compared with MEB control*;

▴ *Statistically significant compared with the other culture filtrate treatment (Mann-Whitney U test, p < 0.05)*.

Focusing on shoot parameters, while no statistically significant differences have been observed in the numbers of leaves, a statically significant difference (*p* < 0.05) in leaf area has been observed comparing *M. polyspora* treatment with both MEB control and *C. globosum* treatment. It is interesting to note that the leaf area values of the plants treated with *C. globosum* culture filtrate resulted to be lower than controls, although it did not result to be statistically significant. Finally, no significant differences were observed among the treatments concerning the indexes of root/shoot ratio and SLA.

### ^1^H-NMR Analysis of Culture Filtrates

The filtrate composition is reported in Supplementary Table S1. ^1^H-NMR analysis of *C. globosum* culture filtrate showed a decrease in all amino acids including leucine, isoleucine, valine, threonine, lysine, tyrosine, phenylalanine, glutamate, and acetate that resulted to be consumed by the fungus and an increase in peptone, ethanol, and fumarate that were released. A slight decrease in glucose, maltose, and fructose was also observed. Conversely, in *M. polyspora* culture filtrate, a high increase in glucose associated with a decrease in maltose was observed. No variation in amino acids except for alanine and lysine was observed. A production of adenosine and guanosine phosphates nucleotides (AXP and GXP, respectively) was also detected.

### ^1^H-NMR-Based Metabolomics of *Cichorium intybus* Leaves and Roots

The inspection of the 600 MHz ^1^H-NMR spectra obtained from hydroalcoholic and chloroformic extracts of chicory leaves and roots revealed the presence of 49 molecules univocally identified. A total of 46 metabolites including amino acids, organic acids, sugars, organic compounds, fatty acids, secondary metabolites, and other compounds were integrated. Only the molecules univocally identified were considered for the study, and their quantification was performed by integration of their NMR signals. Due to the overcrowding of ^1^H-NMR spectra, only those signals that did not overlap with other resonances were considered for integration. Comparing the spectra obtained from leaves and roots, it was possible to observe both qualitative and quantitative differences, while the spectral comparison among the control group and the treatments showed only quantitative differences. Examples of ^1^H-NMR spectra are reported in Supplementary Figures S1–S4, and the table of resonance assignment is reported in Supplementary Table S2. Quantitative analysis of the phytochemical composition of *C. intybus* roots and leaves are reported in Supplementary Tables S3, S4, respectively.

To examine an overview of the whole NMR data set, a preliminary unsupervised PCA was performed in leaves and roots separately. In leaves, PC1 and PC2 components explained 42% of the total variation, and PCA score plot revealed a clustering of the samples according to the treatment with the fungal filtrate (Supplementary Figure S5). In roots, 41% of the total variation was explained by the two main components, with the first and second contributing 24 and 17%, respectively (Supplementary Figure S6).

With the aim of refining the sample grouping observed in the unsupervised PCA model, PLS-DA discriminant analyses were performed to identify the most important metabolites that discriminated the treatments with *C. globosum* and *M. polyspora* separately in leaves and roots.

### Effects of *Chaetomium globosum* Culture Filtrate on the Metabolome of *Cichorium intybus* Leaves and Roots

**Figure 2:**
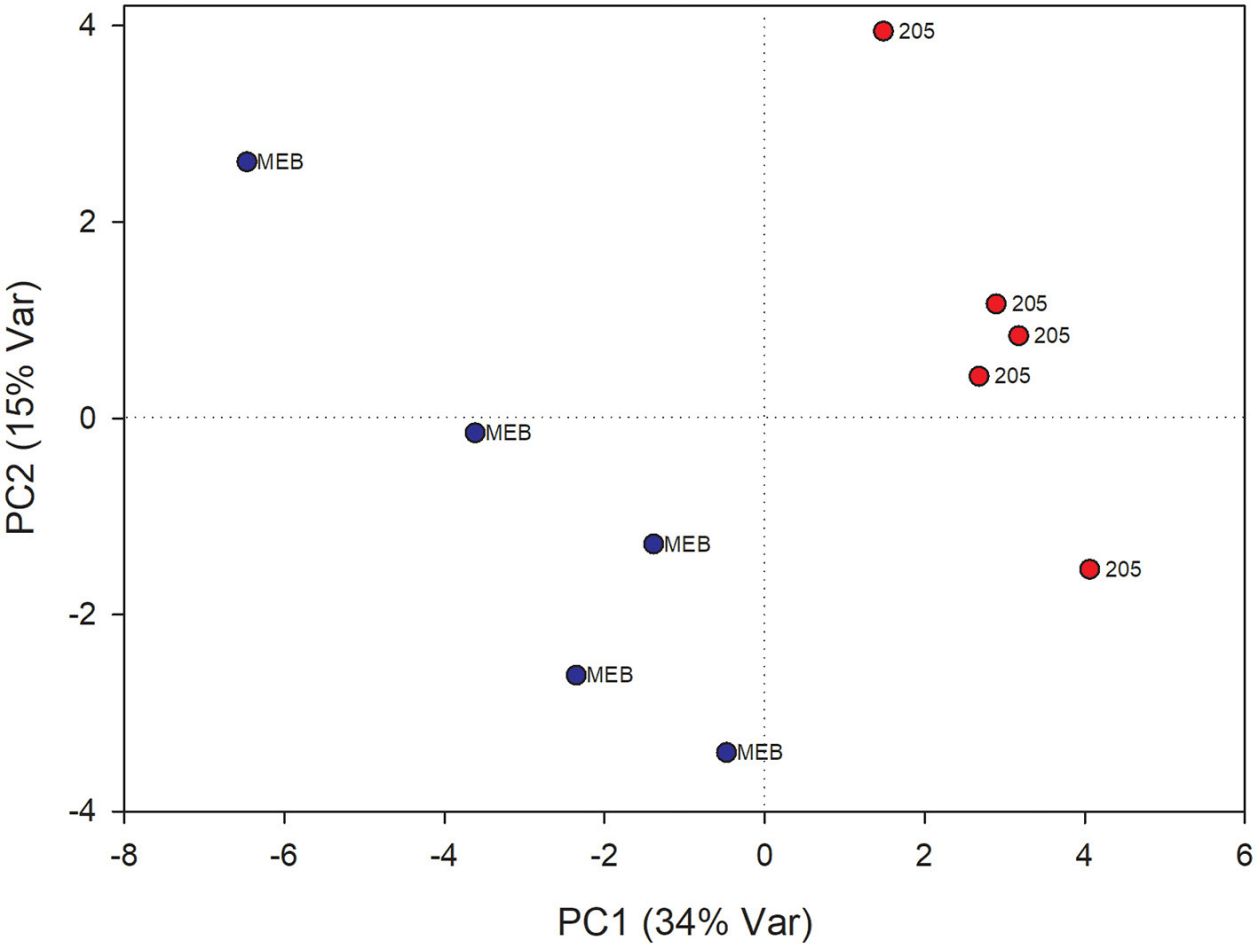
PLS-DA score plot of NMR metabolomics data of chicory leaves extracts of MEB (control) and *C. globosum* (205) groups. Blue dots indicate MEB (control) group and red dots indicate *C. globosum* group.

In leaves treated with *C. globosum* filtrate, PLS-DA analysis provided a model (R2 =0.96; Q2 =0.62) with three discriminant components explaining 34, 76, and 15% of the variance (Figure 2). The corresponding PLS-DA score plot revealed a clear separation of the leaves treated with *C. globosum* filtrate (205) from control samples.

**Figure 3:**
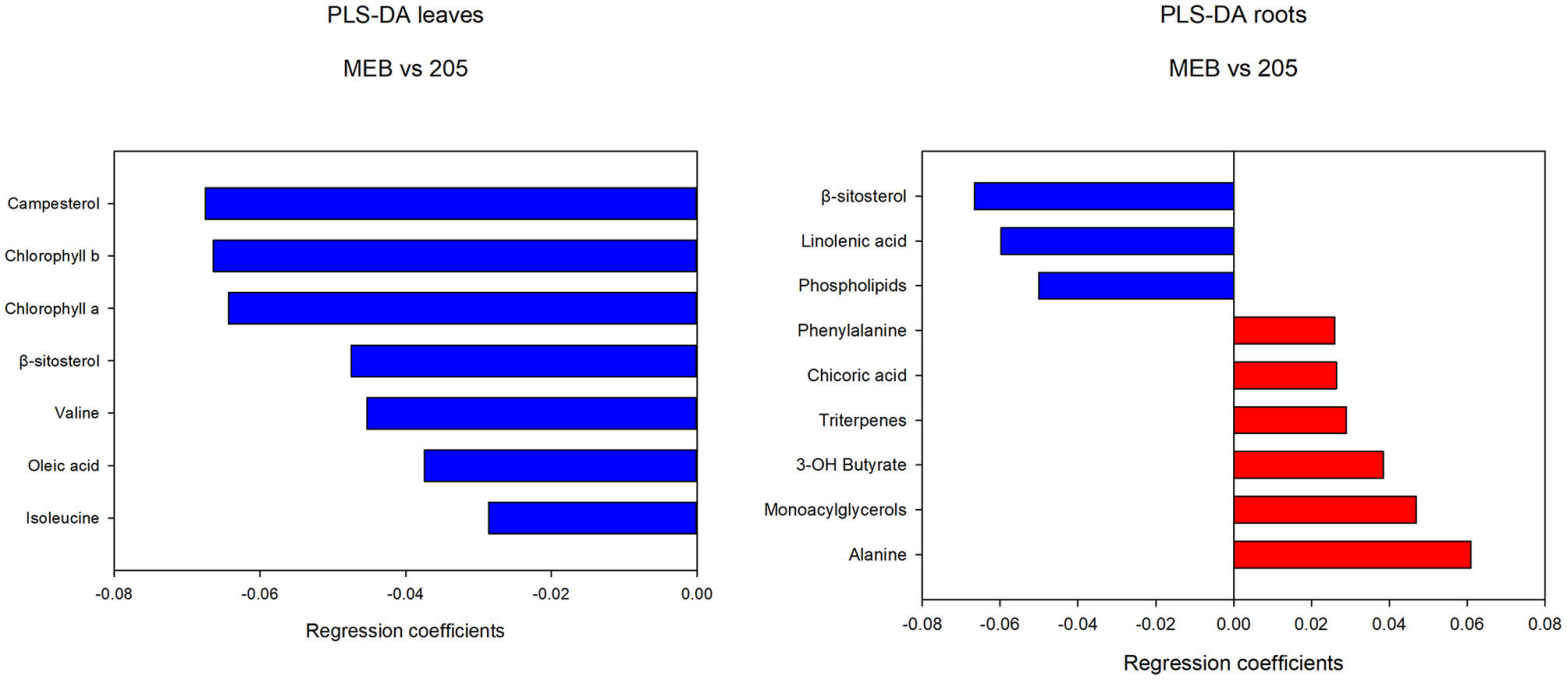
PLS-DA regression coefficients of significantly different metabolites in chicory leaf and root extracts of MEB (control) and *C. globosum* (205).

The levels of campesterol, chlorophyll *a* and *b*, β*-*sitosterol, valine, oleic acid, and isoleucine decreased in *C. globosum* group compared with controls (Figure 3).

**Figure 4:**
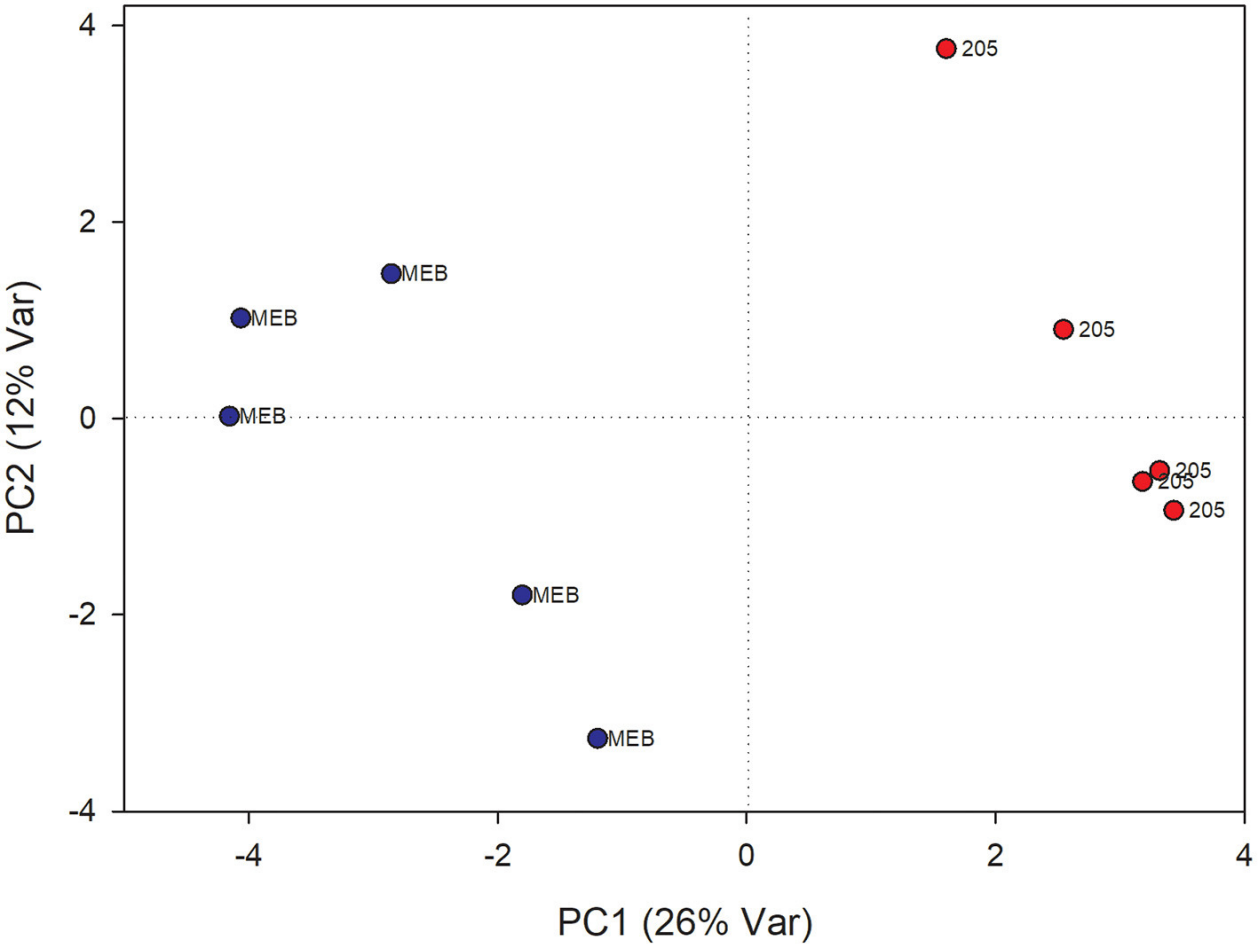
PLS-DA score plot of NMR metabolomics data of chicory roots extracts of MEB (control) and *C. globosum* (205) groups. Blue dots indicate MEB (control) group and red dots indicate *C. globosum* group.

In roots treated with *C. globosum* filtrate, the PLS-DA model showed a clear separation of samples with good descriptive parameters (R2 =0.97; Q2 =0.75) (Figure 4). A significant decrease in β*-*sitosterol, linolenic acid (ω-3), and phospholipids and a significant increase in phenylalanine, chicoric acid, triterpenes, 3-OH-butyrate, monoacylglycerols, and alanine were observed in *C. globosum* compared with control samples (Figure 3).

### Effects of *Minimedusa polyspora* Culture Filtrate on the Metabolome of *Cichorium intybus* Leaves and Roots

**Figure 5:**
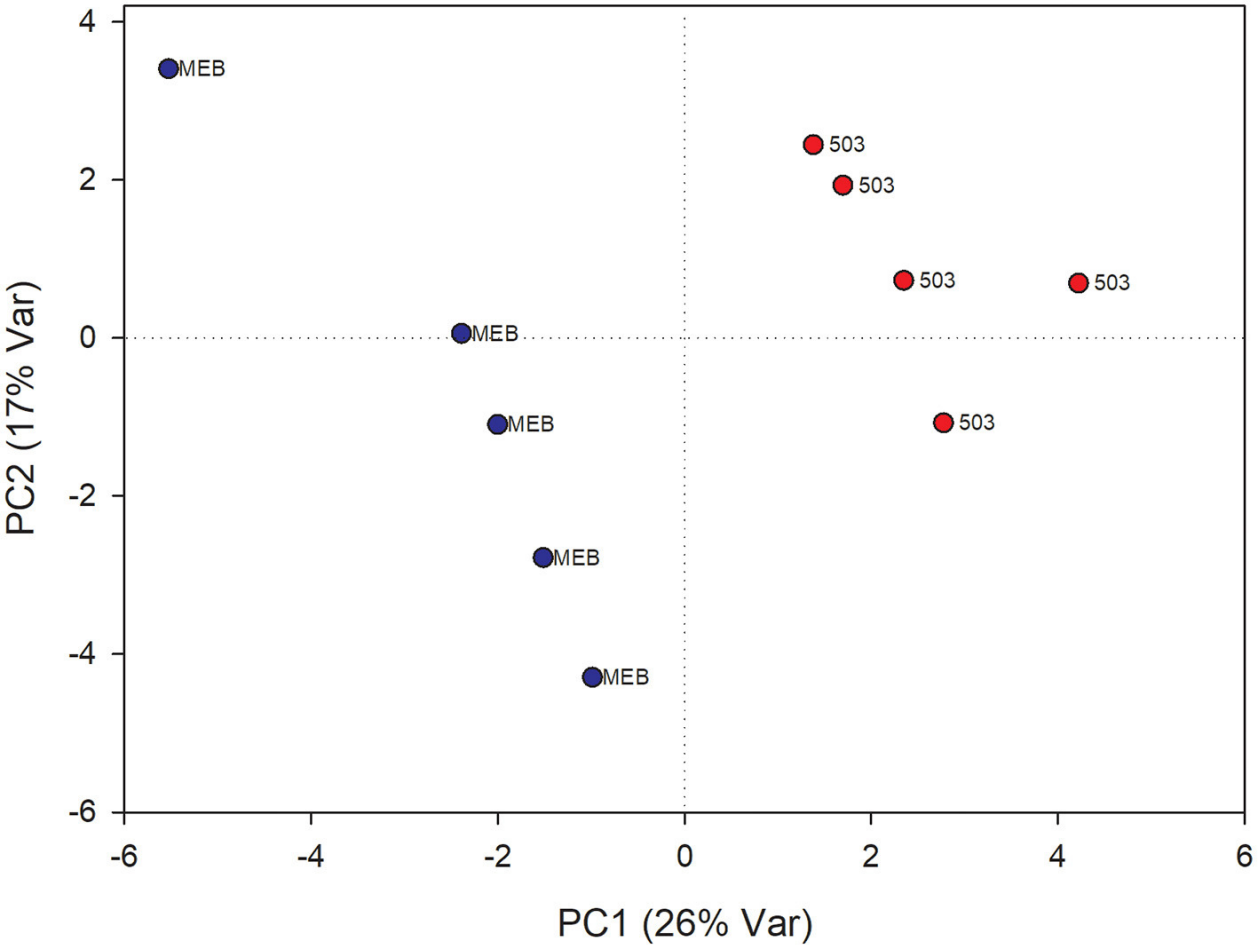
PLS-DA score plot of NMR metabolomics data of chicory leaves extracts of MEB (control) and *M. polyspora* (503) groups. Blue dots indicate MEB (control) group and red dots *M. polyspora* group.

**Figure 6:**
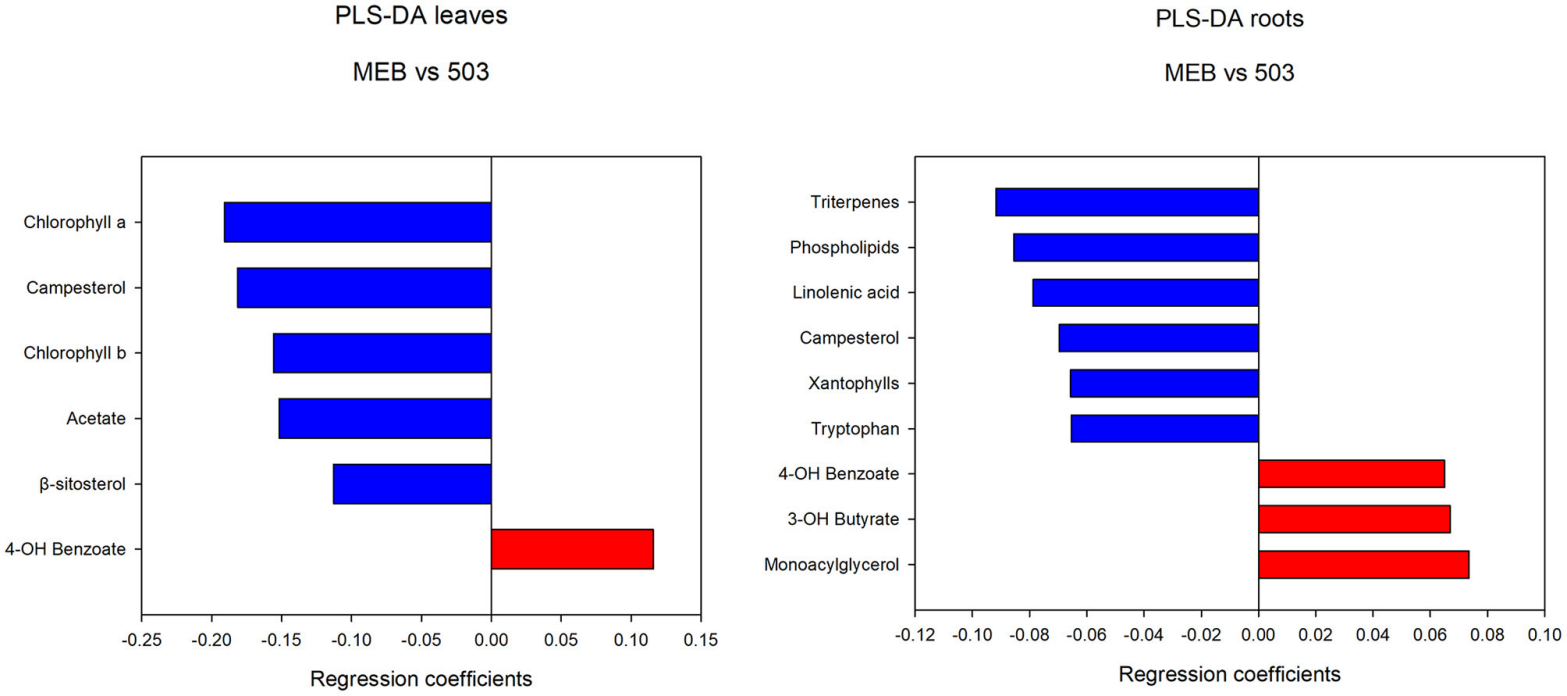
PLS-DA regression coefficients of significantly different metabolites in chicory leaf and root extracts of MEB (control) and *M. polyspora* (503).

**Figure 7:**
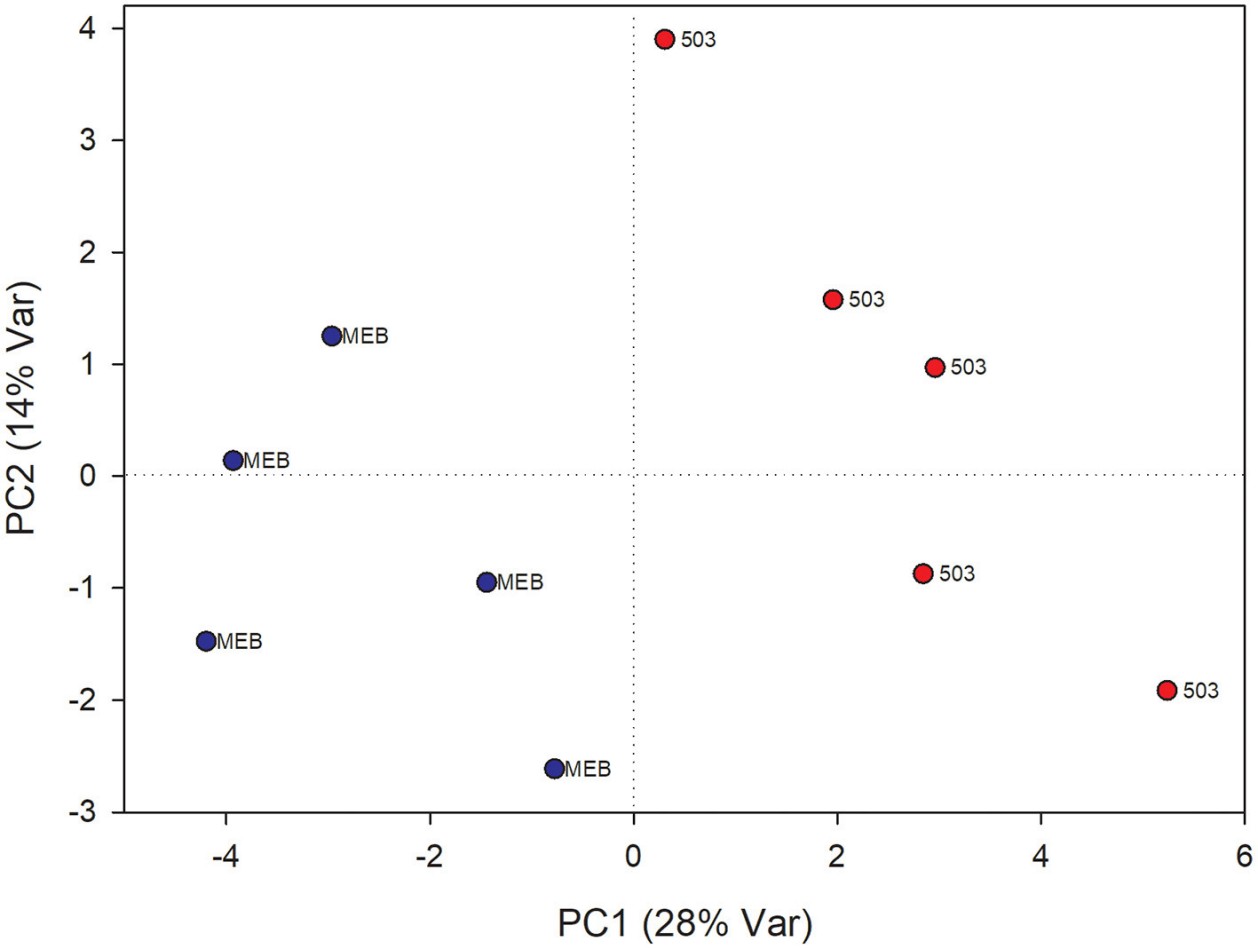
PLS-DA score plot of NMR metabolomics data of chicory roots extracts of MEB (control) and *M. polyspora* (503) groups. Blue dots indicate MEB (control) group and red dots *M. polyspora* group.

The PLS-DA analysis performed to highlight metabolic differences related to *M. polyspora* treatment in *C. intybus* leaves showed an excellent sample separation (R2 = 0.99; Q2 = 0.62) (Figure 5). Similar to *C. globosum* treatment, a significant decrease in chlorophyll *a* and *b*, campesterol, acetate, and β-sitosterol and a significant increase in 4-OH-benzoate were observed in leaves of plants treated with *M. polyspora* filtrate compared with control samples (Figure 6). In the PLS-DA model (R2 = 0.77; Q2 = 0.52) of root samples (Figure 7), discriminant variables such as triterpenes, phospholipids, linolenic acid (ω-3), campesterol, xantophylls, and tryptophan were significantly decreased, while 4-OH-benzoate, 3-OH-butyrate, and monoacylglycerol were significantly increased in roots treated with *M. polyspora* filtrate compared with controls (Figure 6).

## Discussion

### Effects of *Chaetomium globosum* and *Minimedusa polyspora* Culture Filtrates on *Cichorium intybus* Growth Parameters

Fungal culture filtrates have consistently been reported as effective biostimulants. In the environment, fungi due to a complex extracellular metabolism exert their influence on plants also through the release of diffusible metabolites in soils. Therefore, learning from nature, the metabolites released in the culture medium during growth in controlled conditions may be used to simulate these interactions and effectively stimulate plant growth.

In this study, we showed that soil application of *M. polyspora* culture filtrate promotes biomass production, both in shoot and root, and leaf area extension in *C. intybus* plants (Table 1). Considering that no statistically significant differences in the number of leaves were observed, the increase in biomass production seems to be related to the significant increase in leaf area observed. The significant increase in root biomass more specifically occurred in the lateral roots biomass since the taproot biomass did not show differences among the treatments. An increase in lateral roots may have resulted in a more effective exploration of the soil and therefore an increased efficiency in nutrient uptake leading to a higher growth.

In this study, *M. polyspora* is reported for the first time to have a direct plant growth-promoting effect. This effect is in line with what is reported in previous studies on the fungal culture filtrate biostimulant effect. For example, the culture filtrate of *Serendipita indica*, which, like *M. polyspora*, is an anamorphic species belonging to the Basidiomycota phylum, has been widely reported to increase the biomass production and other growth parameters including plant height, leaves length and width, root length and number, and fruit and seeds production in *Helianthus annus* (common sunflower), *C. intybus* (chicory), *Zea mays* (maize), *Bacopa monniera* (water hyssop), and *Nicotiana tabacum* (tobacco) plants (Varma et al., 1999; Bagde et al., 2011, 2013; Rashnoo et al., 2020).

Several other fungal strains, including *Alternaria alternata, Aspergillus fumigatus, Byssochlamys spectabilis, C. globosum, Cladosporium* sp., *Fusarium tricinctum, Fusarium proliferatum, Gibberella* spp., *Penicillium melinii, Penicillium citrinum, Penicillium oxalicum, Penicillium* sp., *Phoma herbarum, Sclerotium rolfsii, Shimizuomyces paradoxus, Trichoderma virens, Trichoderma pseudokoningii*, and *Trichoderma harzianum*, have been reported for their culture filtrates biostimulant effect determining an increase in growth parameters in several plant species including rice, cucumber, chickpeas, wheat, canola, tobacco, and pearl millet (Singh et al., 2003; Khan et al., 2008, 2015; Hamayun et al., 2009, 2010; Hwang et al., 2011; Sung et al., 2011; Rahman et al., 2012; López-Bucio et al., 2015; Murali and Amruthesh, 2015; Bilal et al., 2018; Haruma et al., 2018; Zhai et al., 2018; Zhou et al., 2018; Jahagirdar et al., 2019; Naziya et al., 2019; Baron et al., 2020; Ozimek and Hanaka, 2020; Khalmuratova et al., 2021; Tarroum et al., 2021).

Baroja-Fernández et al. (2021) recently reported the efficacy of *T. harzianum, A. alternata*, and *Penicillium aurantiogriseum* culture filtrates in promoting plant growth of *Capsicum annuum* (peppers). Moreover, the culture filtrates of the three fungal species were found to contain considerable concentrations of glucose and fructose, and a complex mixtures of amino acids, some of which were previously reported to be involved in signaling mechanisms for environmental changes. Similarly, in *M. polyspora* culture filtrate, we detected essential amino acids (e.g., alanine, valine, lysine, and leucine), adenosine and guanosine phosphates nucleotides (AXP and GXP), and glucose at higher concentrations than in MEB (Supplementary Table S1). The higher concentrations of these metabolites may explain the significant variations of growth parameters in treated chicory plants compared with both the controls. Specifically, in *M. polyspora* filtrate, alanine and lysine were higher than the control, while other amino acids (leucine, valine, threonine, glutamate, tyrosine, phenylalanine) occurred in quite similar concentrations. Indeed, fungi can synthesize lysine *de novo* through the so-called α-aminoadipate pathway with α-ketoglutarate as the precursor, while alanine can be formed by transamination from glutamate and pyruvate by glutamate-pyruvate aminotransferase (Smith and Berry, 1976; Zabriskie and Jackson, 2000). Amino acids along with other growth factors present in culture filtrates of some *Fusarium* species have been reported to promote the growth of *Oryza sativa*'s (rice) roots (Ram, 1959). Moreover, the presence of essential amino acids in culture filtrates can confer stress resistance to plants, such as alanine in hypoxic conditions in plants (Podlešáková et al., 2019; Baroja-Fernández et al., 2021). Lysine has been reported to be involved in plant growth and stress resistance, as well. In fact, roots can take up several amino acids, including lysine, and directly incorporate them into new cell biomass and utilize them for respiration (Owen and Jones, 2001). Moreover, foliar application of iron-conjugated lysine on *Brassica napus* (rapeseed) has been reported to increase plant growth, biomass production, chlorophyll content, and essential micronutrients uptake and to reduce oxidative stress enhancing antioxidant enzyme activities in response to chromium stress condition (Zaheer et al., 2020).

Furthermore, the presence of glucose and adenosine and guanosine phosphates nucleotides, as energy sources, can exert a plant growth promotion activity in chicory. In particular, this higher level of glucose compared with MEB may be explained by the extracellular hydrolysis of maltose, leading to the formation of two molecules of glucose, as reported in *Aspergillus niger* through the maltase enzyme (Yuan et al., 2008; Hamad et al., 2015). Indeed, maltose in *M. polyspora* filtrate was almost completely depleted, and it is reasonable to think that this was determined by the release of hydrolytic enzymes, as this cellulolytic strain in different development stages can metabolize polysaccharides, hexoses, and oligosaccharides (Pinzari et al., 2014). Conversely, *C. globosum* culture filtrate in our study did not exert any effect in increasing the biomass production and the other morphological parameter's values. Nevertheless, *C. globosum* and its metabolites have been previously reported as an effective plant growth promoter of *C. annuum* (pepper), *Brassica juncea* (mustard), *Solanum lycopersicum* (tomato), *Pennisetum americanum* (pearl millet), *Z. mays* (maize), and *N. tabacum* (tobacco). In all the abovementioned species, an increase in biomass production was recorded, and, as appropriate, *C. globosum* caused an increase also in various morphological and physiological parameters of growth and development such as shoot growth, plant height, root length, leaf area, chlorophyll content, stomatal conductance, transpiration rate, seed germination, and nutrient uptake (Tarafdar and Gharu, 2006; Abou Alhamed and Shebany, 2012; Khan et al., 2012; Kumar et al., 2021; Singh et al., 2021; Tarroum et al., 2021). Unfortunately, no other studies addressing the effect of *C. globosum* on *C. intybus* are available in the literature, and therefore, it is not possible to determine whether its inefficiency is related to the test conditions in this study or to the specific plant-fungal strain interaction. In fact, a PGPF that results effective in promoting the growth of a given plant species may be less effective or even not present the same beneficial effect at all upon another plant species. Moreover, environmental conditions may also affect the beneficial effect of a PGPF (Hossain et al., 2017).

### Impact of Soil Application of *Chaetomium globosum* and *Minimedusa polyspora* Culture Filtrates on *Cichorium intybus* Metabolome: Common Threads in Leaves and Root Metabolism

Beneficial microorganisms are known to release diffusible substances that promote plant growth. Consistently, soil application of fungal culture filtrates can affect plant metabolism, growth, and yield (Baroja-Fernández et al., 2021).

However, how this treatment acts in plants is largely unknown. In this work, we characterized the responses of chicory (*C. intybus*) plants cultured under greenhouse conditions to soil application of culture filtrates obtained from *C. globosum* (205) and *M. polyspora* (503).

**Figure 8:**
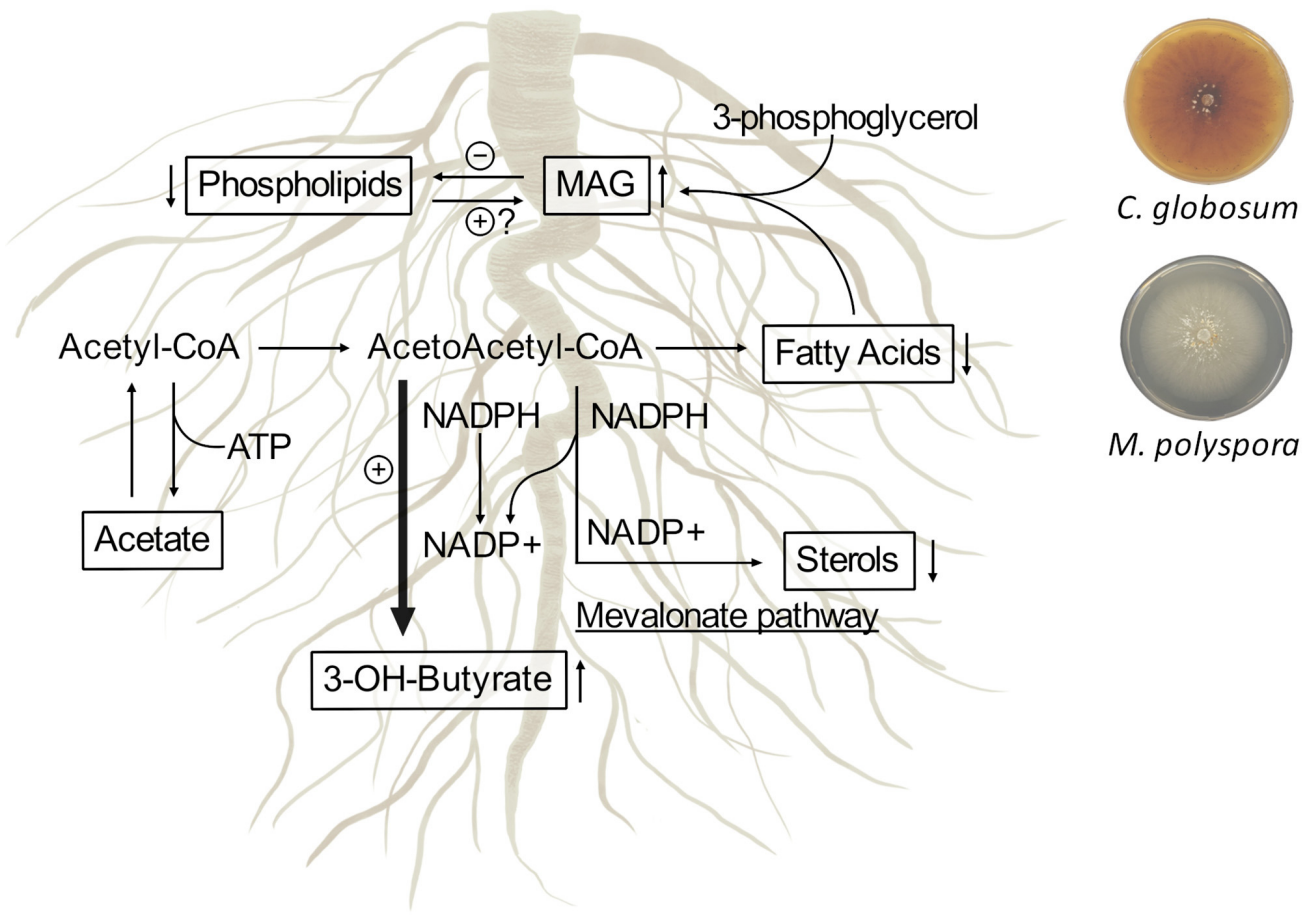
Metabolic network involving 3-OH-butyrate, phospholipids, sterols, and fatty acids occur in *C. intybus* roots after treatment with *C. globosum* (205) and *M. polyspora* (503) culture filtrates. The identified metabolites and their variations are reported in squares.

^1^H-NMR-based metabolomics analysis of *C. intybus* roots after treatment with *C. globosum* and *M. polyspora* culture filtrates revealed common metabolic threads involving the increase of 3-OH-butyrate and monoacylglycerols associated with the decrease of unsaturated fatty acids (UFAs) such as linolenic acid, sterols including campesterol and β-sitosterol, and phospholipids. We outlined the metabolic network involving 3-OH-butyrate, phospholipids, sterols, and fatty acids occurring in *C. intybus* roots after treatment with *C. globosum* (205) and *M. polyspora* (503) (Figure 8).

Differently from animals, in which 3-OH-butyrate is an intermediate metabolite of fatty acids, the physiological importance and the metabolism of 3-OH-butyrate in plants are not fully understood and characterized. Mierziak et al. (2020) demonstrated that 3-OH-butyrate occurs naturally in flax and could act as a regulatory molecule that most likely influences the expression of genes involved in DNA methylation, thereby altering DNA methylation levels. Recent studies showed that plants contain enzymes such as β-ketothiolase (EC 2.3.1.9) and acetoacetyl-CoA-reductase (EC 1.1.1.36), which are involved in the synthesis of 3-OH-butyrate as in bacteria and animals (Xu et al., 1997; Beaudoin et al., 2009; Jin et al., 2012; Tsuda et al., 2016; Mierziak et al., 2021). In plants, biosynthesis of fatty acids and biosynthesis of sterols, which takes place *via* mevalonate pathway in plastids and endoplasmic reticulum, occur from acetyl-CoA and acetoacetyl-CoA through NADPH/NADP^+^ recycling and reductase/synthase enzyme activities. The treatment with *C. globosum* (205) and *M. polyspora* (503) culture filtrates stimulates a common response in *C. intybus* roots involving the synthesis of 3-OH-butyrate through the decrease of the synthesis of fatty acids and sterols, as a mechanism balancing the NADPH/NADP^+^ ratio.

In most plants, the predominant unsaturated fatty acids (UFAs) are three 18-carbon (C18) species, i.e., 18:1 (oleate), 18:2 (linoleate), and 18:3 (α-linolenate) (He et al., 2020). UFAs compounds play multiple crucial roles and are deeply associated with both abiotic and biotic stresses. Besides being membrane ingredients and modulators in glycerolipids, as well as carbon and energy reserve in triacylglycerols (TAGs), C18 UFAs serve as intrinsic antioxidants, precursors of various bioactive molecules [typically the stress hormone jasmonic acid (JA)], and stocks of extracellular barrier constituents such as cutin and suberin (He et al., 2020). The predominant sterols in plants, such as β-sitosterol, campesterol, and stigmasterol, are precursors of a group of plant hormones the brassinosteroids, such as gibberellins and abscisic acid, which regulate plant growth and development (Valitova et al., 2016). Plant lipids, released from the roots into the rhizosphere, facilitate signaling and actively shape the microbiome inhabiting the rhizosphere and the subsequent colonization of their root tissues. Lipids play essential roles as the “chemical language” that facilitates the exchange of resources and modulates the cell responses by inhibiting pathogen attack or enhancing microbial symbiosis. The recruitment of the rhizobiome into the plant vicinity is mediated by rhizodeposits. The release of rhizodeposits comes with a wide variety of substances, such as sugars, amino acids, organic acids, enzymes, growth factors and vitamins, flavanones and purines/nucleotides, and miscellaneous substances. In our study, we observed a decrease in root phospholipids, fatty acids, and sterols that are probably released from roots into the rhizosphere as chemical signals and/or chemotactic attractors to facilitate the recruitment, nutrition, shaping, and tuning of the microbial communities. The perception of these compounds could lead to the stimulation of regulatory or signaling cascades that cause various responses in the microbes. We also observed an increase in monoacylglycerols (MAG) levels. MAG comprise the bulk of oil storage in plant tissues and are involved in many regulatory processes such as cell signaling and intracellular trafficking (Macabuhay et al., 2022).

^1^H-NMR-based metabolomics analysis of *C. intybus* leaves after treatment with *C. globosum* and *M. polyspora* culture filtrates showed the reduction in chlorophylls *a* and *b* content not associated with leaf greening decrease during the plant development and/or treatment with fungal culture filtrates. Dynamic control of chlorophyll levels determined by the relative rates of chlorophyll anabolism and catabolism processes, that largely occur in chloroplasts, ensures optimal photosynthesis and plant fitness. The accumulation of adequate amounts of chlorophyll is therefore vital for plants to establish photosynthetically active chloroplasts during leaf greening. Furthermore, optimized chlorophyll degradation is not only essential for the detoxification of free chlorophyll released but also indispensable for the remobilization of nutrients during leaf development and senescence. Thus, efficient photosynthesis, plant fitness, and yield are critically dependent on the dynamic regulation of chlorophyll levels in response to various developmental and environmental cues (Wang et al., 2020).

### *Chaetomium globosum* Culture Filtrate Triggers Phenylpropanoid Pathway in *Cichorium intybus* Roots: Biosynthesis of Chicoric Acid

**Figure 9:**
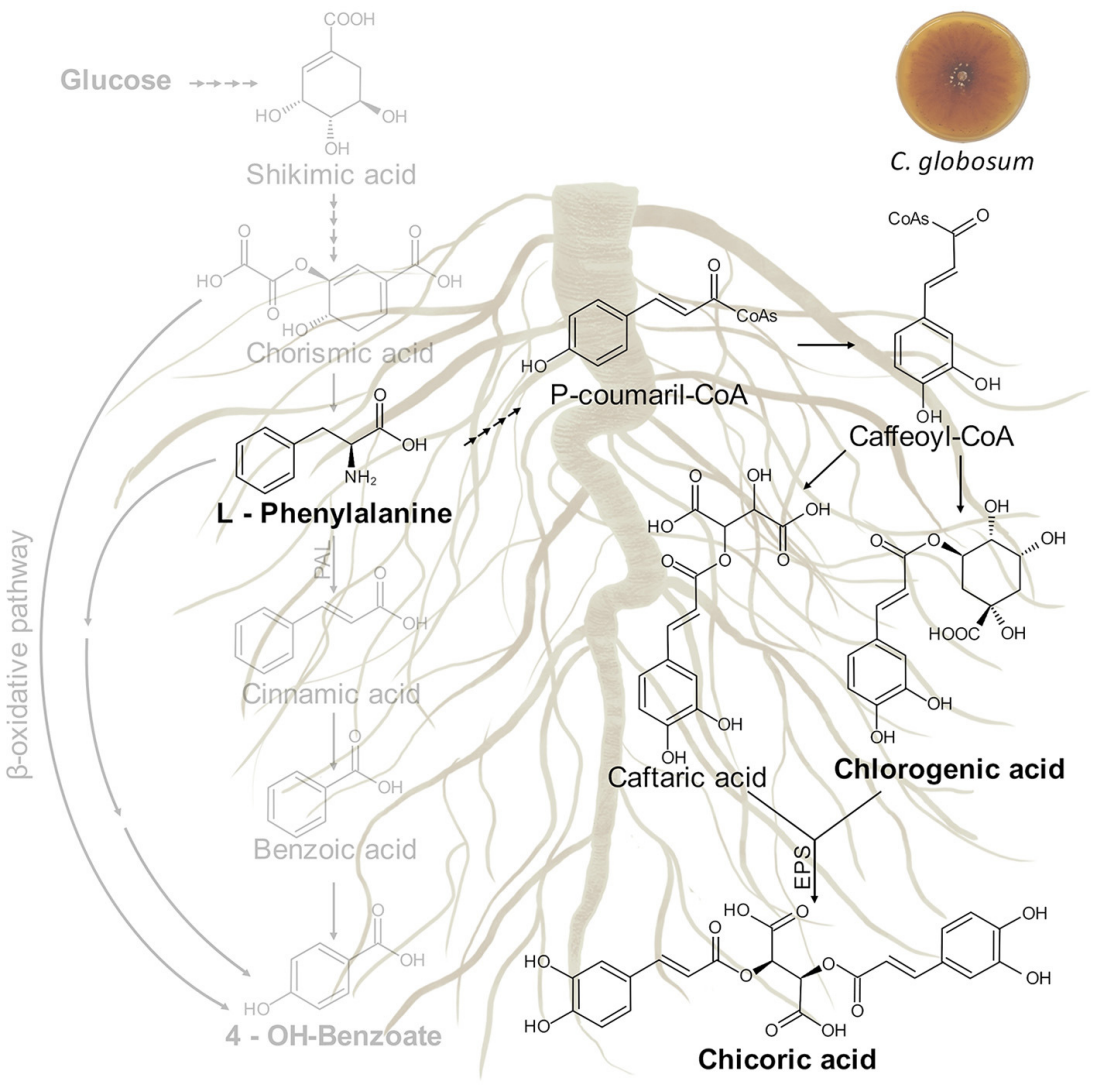
Putative metabolic pathway involved in chicoric acid biosynthesis in *C. intybus* roots. In bold are reported the identified metabolites. EPS: acyltransferase.

In our study, the treatment of *C. intybus* plants with *C. globosum* culture filtrate triggered the phenylpropanoid pathway through an increase of phenylalanine and chicoric acid in roots. The biosynthetic pathway of chicoric acid in plants is putative and still not well known, although it is generally understood to form *via* shikimic acid/phenylpropanoid pathway as other phenolics, analogous to the conjugation of caffeic acid derivatives of rosmarinic acid or chlorogenic acid (Legrand et al., 2016; Peng et al., 2019). Putative metabolic pathway involved in chicoric acid biosynthesis in *C. intybus* roots is shown in Figure 9.

The entry point is the aromatic amino acid phenylalanine (Phe) arising from the shikimate pathway. Deamination of Phe by Phe ammonia lyase (PAL) leads to cinnamic acid. Cinnamate-4-hydroxylase and 4-coumarate coenzyme A (CoA) ligase (4CL) generate p-coumaroyl-CoA from cinnamic acid. Thereafter, hydroxycinnamoyl transferases (HCTs) convert the CoA-thioester to coumaroyl quinate or coumaroyl shikimate which is subsequently hydroxylated by p-coumarate-30-hydroxylase to form the caffeoyl derivatives. A recent study carried out on purple coneflower highlighted two types of acyltransferases distributed in distinct subcellular compartments and involved in the biosynthesis of chicoric acid. In the cytosol, the BAHD acyltransferase family including a tartaric acid hydroxycinnamoyl transferases (HTT) and a quinate hydroxycinnamoyl transferases (HQT) use caffeoyl CoA from phenylpropanoid metabolism as an acyl donor to synthesize caftaric acid and chlorogenic acid, respectively (D'Auria, 2006). Both products are then transported into the vacuole where CAS, a specialized serine carboxypeptidase-like (SCPL) acyltransferase, uses chlorogenic acid as its acyl donor and transfers the caffeoyl group to caftaric acid to generate chicoric acid (Fu et al., 2021).

For millennia, humans have used plant specialized metabolites as herbal medicines. Chicoric acid is a very promising natural compound, which occurs in a variety of plant species such as *C. intybus, Echinacea purpurea* L. (purple coneflower), *Ocimum basilicum* L. (basil), *Lactuca sativa* L. (lettuce), *Taraxacum officinale* (dandelion), *Cucurbita pepo* L. (squash), and *Borago officinalis* L. (borage) (Lee and Scagel, 2013). According to the literature data, chicoric acid plays an important role in plant defense against different diseases caused by viruses, bacteria, fungi, nematodes, and insects (Nishimura and Satoh, 2006; Cheynier et al., 2013).

### *Minimedusa polyspora* Culture Filtrate Triggers Phenylpropanoid Pathway in *Cichorium intybus* Leaves and Roots: Biosynthesis of 4-OH-Benzoate

**Figure 10:**
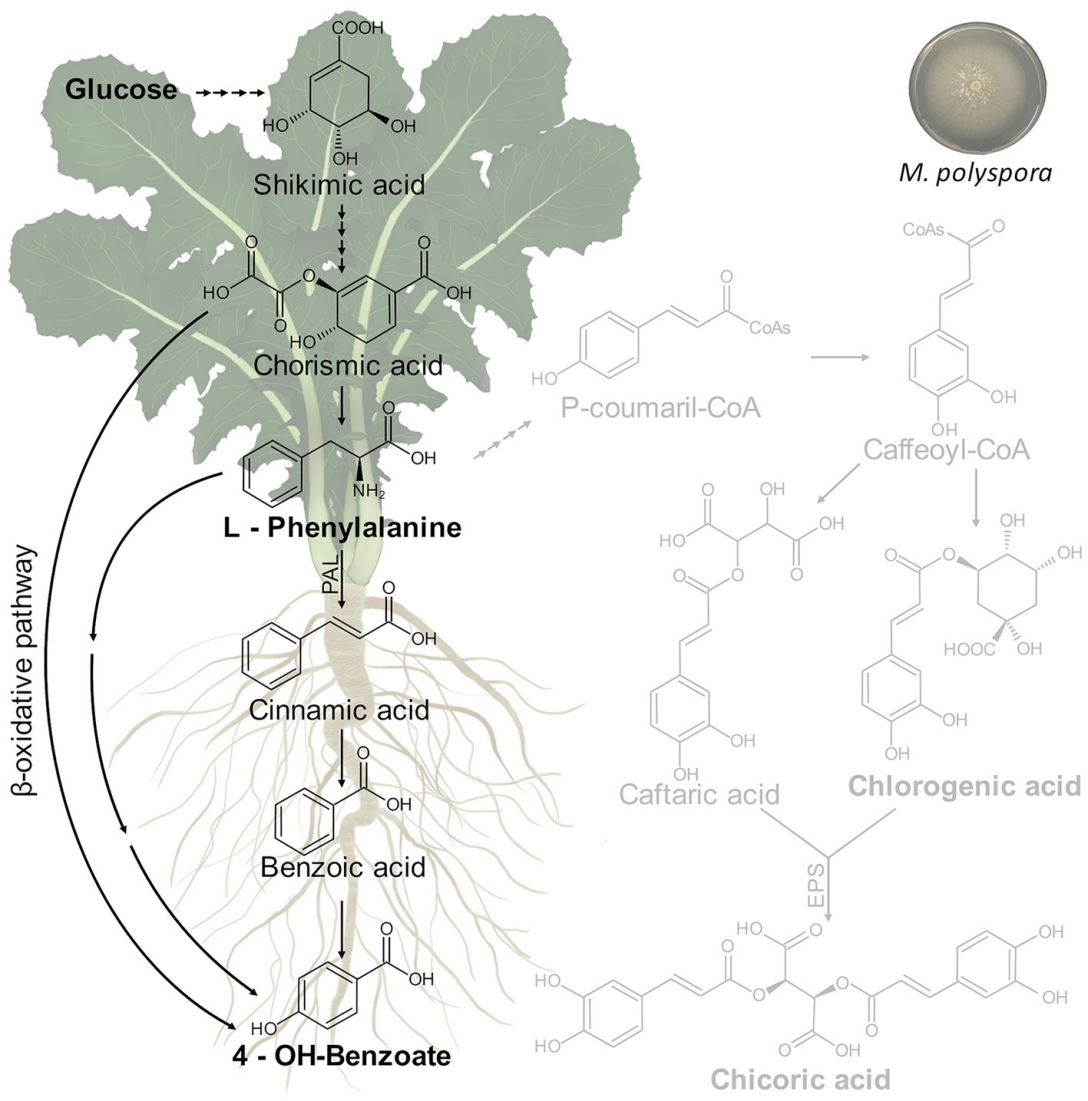
Putative metabolic pathway involved in 4-OH-benzoate biosynthesis in *C. intybus* plant (root and shoot) after treatment with *M. polyspora* (503) culture filtrate.

As reported above, the treatment of *C. intybus* plants with *M. polyspora* culture filtrate determined significant variations of growth parameters in chicory plants including the dry weight of the total plant, shoot, total roots, lateral roots, and leaf area that increased compared with control plants. A relationship between growth parameters and metabolic changes was observed. In particular, *M. polyspora* culture filtrate triggered the phenylpropanoid pathway through an increase of 4-OH-benzoate, which is generated from aromatic amino acids produced *via* the shikimate pathway (Figure 10).

The shikimic acid is converted into L-phenylalanine through a chorismic acid intermediate. Thus, the L-phenylalanine is converted into p-coumaric, salicylic, and p-hydroxybenzoic acids, which serve as precursors for other derivatives of phenolic acids. It is thought that hydroxybenzoic acids can be produced from structurally analogous hydroxycinnamic acids in coenzyme A (CoA)-dependant (β-oxidative) or CoA-independent (non-β-oxidative) pathways or the combination of both of them, which have been determined as occurring in peroxisomes and mitochondria (Widhalm and Dudareva, 2015). Benzoic acids serve as precursors for a wide variety of essential compounds and natural products playing crucial roles in plant fitness and in defense response activation. In this context, 4-OH-benzoate showed *in vitro* antifungal effects on *Eutypa lata* growth (Amborabé et al., 2002).

## Final Remarks

In this study, we investigated the biostimulant effect of fungal culture filtrates obtained from *M. polyspora* and *C. globosum* on *C. intybus* growth and metabolism. For the first time, we showed that *M. polyspora* filtrate exerted a direct plant growth-promoting effect, since the application in soil promoted an increase of biomass, both in shoot and root, and of the leaf area in *C. intybus* plants. Conversely, no significant effect on morphological traits and biomass yield was observed in *C. intybus* plants treated with *C. globosum* culture filtrate.

Based on ^1^H-NMR metabolomics data, differential metabolites and their related metabolic pathways were described. We highlighted that the treatment with *C. globosum* and *M. polyspora* culture filtrates stimulated a common response in *C. intybus* roots involving the synthesis of 3-OH-butyrate through the decrease of the synthesis of fatty acids and sterols, as a mechanism balancing the NADPH/NADP^+^ ratio. Interestingly, the two fungal culture filtrates differently affected the phenylpropanoid pathway in *C. intybus* plants: the treatment with *C. globosum* culture filtrate triggered the phenylpropanoid pathway through an increase of phenylalanine and chicoric acid in roots, whereas *M. polyspora* culture stimulated an increase of 4-OH-benzoate. Chicoric acid, whose biosynthetic pathway in chicory plant is putative and still not well known, is a very promising natural compound playing an important role in plant defense. On the contrary, benzoic acids serve as precursors for a wide variety of essential compounds playing crucial roles in plant fitness and defense response activation. To the best of our knowledge, this is the first study that shows the biostimulant effect of *C. globosum* and *M. polyspora* culture filtrates on *C. intybus* growth and metabolome, increasing the knowledge on fungal bioresources for the development of biostimulants.

## Data Availability Statement

The raw data supporting the conclusions of this article will be made available by the authors, without undue reservation.

## Author Contributions

AP, VS, AC, EB, FS, AM, and GP contributed to the conception and design of the study. AP acquired the fundings. VS, AC, EB, FS, and OG performed the research. AP, AM, and GP supervised the research and reviewed and edited the manuscript. VS, AC, EB, and FS performed the statistical analysis and wrote the original draft of the manuscript. VS prepared the figures. EB and FS prepared the graphs. All authors have read and agreed to the submitted version of the manuscript.

## Funding

This research was funded by the Sapienza University of Rome, Grant Number RG118164361EBAAA.

## Conflict of Interest

The authors declare that the research was conducted in the absence of any commercial or financial relationships that could be construed as a potential conflict of interest.

## Publisher's Note

All claims expressed in this article are solely those of the authors and do not necessarily represent those of their affiliated organizations, or those of the publisher, the editors and the reviewers. Any product that may be evaluated in this article, or claim that may be made by its manufacturer, is not guaranteed or endorsed by the publisher.
