# Supplementary material for: Biostimulant Effects of Chaetomium globosum and Minimedusa polyspora Culture Filtrates on Cichorium intybus Plant: Growth Performance and Metabolomic Traits
Source: Front Plant Sci. 2022 May 12;13:879076. doi: 10.3389/fpls.2022.879076 (PMC9134003; doi:10.3389/fpls.2022.879076)
Supplement: Supplementary file 1 [file Data_Sheet_1.docx]

**Biostimulant effects of *Chaetomium globosum* and *Minimedusa polyspora* culture filtrates on Cichorium intybus plant: growth performance and metabolomic traits**

Veronica Spinelli^1^, Elisa Brasili^1,2*^, Fabio Sciubba^1,2^, Andrea Ceci^1^, Ottavia Giampaoli^1,2^, Alfredo Miccheli^1,2^, Gabriella Pasqua^1,2^, Anna M. Persiani^1^.

* Corresponding Author

^1^Department of Environmental Biology, Sapienza University of Rome, P.le Aldo Moro 5, 00185 Rome, Italy;

^2^NMR-Based Metabolomics Laboratory (NMLab), Sapienza University of Rome, P.le Aldo Moro 5, 00185 Rome, Italy;

Supplementary Material

**Supplementary Table 1.** Filtrate composition assessed by NMR spectroscopy

| Molecule | Amount (mg/100 ml) | | |
| --- | --- | --- | --- |
|  | MEB Control | *Chaetomium globosum* 205 filtrate | *Minimedusa polyspora* 503 filtrate |
| Leucine | 1.01 | 0.00 | 1.34 |
| Isoleucine | 0.84 | 0.00 | 0.65 |
| Valine | 1.00 | 0.00 | 1.16 |
| Threonine | 1.72 | 0.00 | 1.61 |
| Peptone (eq. Lys) | 5.99 | 12.58 | 0.93 |
| Alanine | 1.90 | 1.80 | 4.27 |
| Glutamate | 0.77 | 0.00 | 0.47 |
| GABA | 1.29 | 0.00 | 0.98 |
| Lysine | 2.93 | 0.00 | 5.16 |
| Tyrosine | 0.72 | 0.00 | 0.61 |
| Phenylalanine | 1.68 | 0.00 | 1.89 |
| Acetate | 0.84 | 0.00 | 0.04 |
| Fumarate | 0.00 | 1.33 | 0.00 |
| Formate | 0.35 | 0.00 | 0.00 |
| Maltose | 1266.79 | 771.54 | 6.60 |
| Glucose | 149.65 | 101.69 | 1040.87 |
| Fructose | 2.77 | 1.63 | 2.13 |
| Ethanol | 0.98 | 17.20 | 6.26 |
| Choline | 0.40 | 0.00 | 0.57 |
| AXP | 0.00 | 0.00 | 1.88 |
| GXP | 0.00 | 0.00 | 1.56 |

**Supplementary Figure 1:** ^1^H spectrum of hydroalcoholic extract of chicory roots.

**Supplementary Figure 2:** ^1^H spectrum of chloroform extract of chicory roots.

 **Supplementary Figure 3:** ^1^H spectrum of hydroalcoholic extract of chicory leaves.

**Supplementary Figure 4:** ^1^H spectrum of chloroform extract of chicory leaves.

**Supplementary Table 2.** Table of resonance assignment

| **Compound** | **Assignment** | **^1^H δ (ppm)** | **Multiplicity** | **^13^C δ (ppm)** | **Portion** |
| --- | --- | --- | --- | --- | --- |
| **Organic acids** | | | | | |
| **Acetic acid (AA)** | **CH_3_** | **1.92** | **s** | 25.98 | L, R |
| **3-hydroxybutyric acid (3HBA)** | γ−CH_3_  α,α’-CH  β−CH | 1.19  2.30-2.40  4.15 | d  dd  m | 24.5  49.12  69.01 | R |
| **Ascorbic acid (AscA)** | CH_2_-2’  CH-1’  **CH-5** | 3.77  3.98  **4.49** | m  m  **d** | 63.6  69.41  77.12 | L |
| **Caffeic Acid (CafA)** | CH-1  CH-2  CH-3  CH-4  **CH-5** | 7.19  7.07  6.96  7.31  **6.27** | d  d  dd  d  **d** | 117.15  124.122  118.85  143.34  124.32 | L |
| **Chicoric Acid**  **(ChA)** | CH-1’, 2’  CH-8  **CH-7**  CH-2  CH-5  CH-6 | 5.54  6.50  **7.72**  7.22  6.96  6.15 | s  d  **d**  d  dd  d | 54.23  115.95  117.56  118.90  117.81  149.59 | L, R |
| **Citric acid (CA)** | **α,γ-CH**  α’,γ’-CH | **2.67**  2.71 | **d**  d | 44.77  44.77 | L |
| **Chlorogenic acid**  **(CGA)** | CH_2_-2’  CH-3’  CH-4’  CH-5’  **CH-8**  CH-7  CH-2  CH-5  CH-6 | 2.02,2.17  5.33  3.88  4.23  **6.39**  6.94  7.12  7.19  7.65 | m  m  dd  m  **d**  d  dd  d  d | 40.11  40.14  75.43  72.93  73.82  117.56  118.90  117.81  149.59 | L, R |
| **Formic acid (FA)** | **CH** | **8.46** | **s** | 171.90 | L, R |
| **Fumaric acid (FumA)** | **CH=CH** | **6.51** | **s** | 137.94 | L, R |
| Lactic acid (LA) | CH_3_  CH | 1.32  4.11 | d  q | 22.95  77.04 | L, R |
| **Malic acid (MA)** | **α- CH**  β.β’- CH | **4.31**  2.38,2.69 | **dd**  dd | 69.33  40.86 | R |
|  |  |  |  |  |  |
| **p-Hydroxybenzoate (pHBA)** | **CH-2,6**  CH-3,5 | **7.80**  6.84 | **d**  d | 132.96  116.21 | L, R |
| Succinic acid (SA) | 2 CH_2_ | 2.39 | s | 36.31 | L, R |
| **Amino acids** | | | | |  |
| **Alanine (Ala)** | **β-CH_3_**  α- CH | **1.49**  3.80 | **d**  q | 19.05  53.56 | L, R |
| Aspartic acid (Asp) | β’-CH  β-CH  α-CH | 2.68  2.72  3.91 | dd  dd  m | 39.31  39.31  55.09 | L, R |
| **Asparagine (Asn)** | β’-CH  **β-CH**  α-CH | 2.86  **2.89**  4.01 | dd  **dd**  m | 37.44  37.44  54.09 | L, R |
| **Glutamate (Glu)** | γ- CH_2_  **β,β-CH_2_**  α-CH | 2.07  **2.35**  3.78 | m  **m**  m | 29.25  37.12  58.01 | L, R |
| **Glutamine (Gln)** | γ- CH_2_  **β,β-CH_2_**  α-CH | 2.11  **2.45**  3.81 | m  **m**  m | 29.31  34.02  57.19 | L, R |
| **Isoleucine (Ile)** | δ-CH_3_  **γ-CH_3_**  γ’-CH  γ’’-CH  β-CH  α-CH | 0.95  **1.02**  1.25  1.49  1.99  3.69 | t  **d**  m  m  m  m | 13.85  17.38  27.01  27.01  38.71  63.04 | L, R |
| **Leucine (Leu)** | **δ,δ’- CH_3_**  γ-CH  β-CH_2_  α-CH | **0.97**  1.72  1.73  3.74 | **m**  m  m  m | 23.85, 24.59  26.81  42.60  56.21 | L, R |
| **Lysine (Lys)** | δ- CH_2_  γ- CH_2_  β- CH_2_  **δ- CH_2_**  α- CH_2_ | 1.47  1.69  1.95  **3.03**  3.78 | m  m  m  **t**  t | 28.41  30.51  43.27  38.12  57.32 | L |
| **γ-aminobutyric acid (GABA)** | **β-CH_2_**  γ-CH_2_  α-CH_2_ | **1.95**  2.30  3.01 | **t**  m  t | 26.38  37.06  42.21 | L, R |
| **Phenylalanine (Phe)** | CH-2,6  CH-4  **CH-3,5**  β-CH_2_  α-CH | 7.32  7.38  **7.42**  3.27  3.98 | d  d  **d**  m  dd | 130.3  128.6  130.3  37.1  56.8 | L, R |
| **Pyroglutamic acid (PyrA)** | γ- CH_2_  **β,β-CH_2_**  α-CH | 2.01  **2.45**  4.16 | m  **m**  m | 33.25  28.32  60.91 | R |
| **Threonine (Thr)** | **γ-CH_3_**  α-CH  β-CH | **1.33**  3.60  4.27 | **d**  m  m | 22.15  63.46  68.94 | L, R |
| **Tryptophan (Trp)** | CH-5  CH-6  **CH-7**  CH-4 | 7.20  7.27  **7.53**  7.73 | t  t  **d**  d | 124.9  127.9  114.7  121.2 | R |
| **Tyrosine (Tyr)** | CH-2,6  **CH-3,5**  β-CH_2_  α-CH | 7.22  **7.08**  3.15  3.93 | d  **d**  dd  dd | 130.0  117.0  37.1  56.8 | L, R |
| **Valine (Val)** | γ-CH_3_  **γ’-CH_3_**  β-CH  α-CH | 0.99  **1.05**  2.29  3.62 | d  **d**  m  m | 19.41  20.75  31.89  63.36 | L, R |
| **Carbohydrates** | | | | |  |
| **Amylose (Amy)** | **CH-1**  CH-2  CH-3  CH-4  CH-5  CH_2_-6 | **5.11**  3.33  3.37  3.42  3.66  3.63, 3.70 | **d**  m  m  m  m  m | 99.10  72.49  77.84  70.67  73.52  66.97 | L, R |
| **α-Glucose (α-G)** | **CH-1**  CH-2  CH-3  CH-4  CH-5  CH_2_-6 | **5.25**  3.55  3.72  3.42  3.84  3.73, 3.90 | **d**  m  m  m  m  m | 93.10  72.49  73.84  70.67  72.52  96.97 | L, R |
| **β-Glucose (β-G)** | **CH-1**  CH-2  CH-3  CH-4  CH-5  CH_2_-6 | **4.69**  3.26  3.50  3.42  3.48  3.74, 3.91 | **d**  m  m  m  m  m | 96.97  75.17  76.84  70.70  74.57  61.80 | L, R |
| **Sucrose (S)** | **GLC CH-1**  CH-2  CH-3  CH-4  CH-5  CH_2_-6  FRU CH_2_-1’  C-2  CH-3’  CH-4’  CH-5’  CH_2_-6 | **5.42**  3.59  3.79  3.48  3.85  3.82  3.69  \  4.22  4.06  3.90  3.82 | **d**  m  m  m  m  m  m  \  m  m  m  m | 93.22  72.11  73.54  70.26  73.38  61.18  62.44  104.85  77.45  75.04  82.44  63.38 | L, R |
| **Lipids & Sterols** | | | | |  |
| **Linoleic acid (n-6 FA)** | CH_3_  n-CH_2_  CH_2_-CH=CH  CH=CH  **=CH-CH_2_-CH=**  CH_2_-CH_2_-CO_2_^-^  CH_2_-CO_2_^-^ | 0.86  1.36  2.04  5.37  **2.76**  2.06  2.31 | t  m  m  m  **t**  m  t | 14.06  29.37  29.45  130.29; 128.45  25.68  24.75  34.05 | L, R |
| **Linolenic acid (n-3 FA)** | CH_3_  n-CH_2_  CH_2_-CH=CH  CH=CH  **=CH-CH_2_-CH=**  CH_2_-CH_2_-CO_2_^-^  CH_2_-CO_2_^-^ | 0.96  1.36  2.04  5.37  **2.78**  2.06  2.31 | t  m  m  m  **m**  m  t | 14.14  29.37  29.45  130.29; 128.45  25.83  24.75  34.05 | L, R |
| **β-Sitosterol (β-ST)** | CH_2_-1  CH_2_-2  CHOH-3  CH_2_-4  CH-6  CH_2_-7  CH-8  CH-14  CH_2_-15  CH_2_-16  **CH_3_-18**  CH_3_-25 | 1.08, 1.85  1.51, 1.84  3.52  2.28  5.34  1.52, 1.98  1.46  0.99  1.57  1.26, 1.85  **0.68**  1.01 | m  m  m  m  m  m  m  m  m  m  **s**  s | 37.19  31.50  71.81  42.37  121.79  31.98  31.78  56.74  24.25  28.37  12.20  19.12 | L, R |
| **Campsterol (Camp)** | CH_2_-1  CH_2_-2  CHOH-3  CH_2_-4  CH-6  CH_2_-7  CH-8  CH-14  CH_2_-15  CH_2_-16  **CH_3_-18**  CH_3_-25 | 1.08, 1.85  1.51, 1.84  3.52  2.28  5.34  1.52, 1.98  1.46  0.99  1.57  1.26, 1.85  **0.70**  1.01 | m  m  m  m  m  m  m  m  m  m  **s**  s | 37.19  31.50  71.81  42.37  121.79  31.98  31.78  56.74  24.25  28.37  12.21  19.12 | L, R |
| **Miscellaneous Metabolites** | | | | |  |
| **Adenosine phosphate (AXP)** | CH-17’  CH-17  CH-5  CH-4  **CH-2**  CH-7 | 4.18  4.24  4.36  4.58  **5.92**  8.12 | ddd  ddd  m  dd  **d**  s | 67.40  67.40  73.10  76.35  89.33  140.39 | L, R |
| **Choline (Chn)** | **N(CH_3_)_3_** | **3.20** | **S** | 56.70 | L, R |
| **7-Hydroxycoumarin (HCou)** | CH-3  CH-4  CH-5  CH-6  CH-8 | 6.34  **7.67**  7.36  7.09  7.13 | d  **d**  d  dd  d | 115.45  143.21  127.55  125.24  117.03 | R |
| **Trigonelline (Trg)** | N-CH_3_  CH4  CH3,5  **CH1** | 4.42  8.07  8.82  **9.11** | s  m  m  **s** | 51.1  130.4  148.5  148.1 | L, R |
| **Uracile (Ur)** | **CH-5**  CH-6 | **5.92**  7.85 | **d**  d | 103.7  146.3 | L, R |
| **Monoacylglycerol (MAG)** | **CH_2_**  CH_2_  CH | **3.65-3,55**  4.05-4.15  3.82 | **dd**  dd  m | 65.45  70.32  75.12 | L, R |
| **Phospholipids (PP)** | CH  **2CH_2_** | **5.13-5.21**  4.15-4.29 | bm  dd | 77.45  68.23 | L, R |
| **Triglycerids (TG)** | CH  **2CH_2_** | **5.33**  4.15-4.29 | m  dd | 74.64  65.93 | A |
| **Triterpenes (TP)** | CH-5  CH-9  CH_2_-11  CH-12  **CH-18**  CH_2_-22 | 0.87  1.65  1.94  5.49  **2.54**  1.97 | m  t  m  t  **d**  t | 55.19  48.34  23.9  125.71  53.22  37.43 | L, R |
| **Carotenoids (Crt)** | CH_2_-2,2’  CH_2_-3,3’  CH_2_-4,4’  CH-7,7’  CH-8,8’  CH-10,10’  **CH-11,11’**  CH-12,12’  CH-14,14’  CH-15,15’  CH_3_-16,16’,17,17’  CH_3_-18,18’  CH_3_-19,19’ | 1.47  1.62  2.02  6.15  6.14  6.14  **6.68**  6.35  6.25  6.63  1.03  1.72  1.97 | m  m  m  d  d  d  **m**  d  d  m  s  s  s | 39.62  19.27  33.18  126.68  137.78  130.88  125.04  137.26  132.45  130.02  29.01  21.77  12.81 | L |
| **Xanthophyl (Xant)** | CH-2,2’  CH-3,3’  CH-4  CH-4’  CH-7,7’  CH-8,8’  CH-10,10’  CH-11,11’  CH-12,12’  CH-14,14’  **CH-15,15’**  CH_3_-16,16’,17,17’  CH_3_-18,18’  CH_3_-19,19’  CH_3_-20,20’ | 1.47  1.62  2.02  5.45  6.15  6.14  6.14  6.68  6.35  6.25  **6.83**  1.03  1.72  1.97  1.96 | m  m  m  bs  d  d  d  m  d  d  **m**  s  s  s  s | 39.62  19.27  33.18  123.18  126.68  137.78  130.88  125.04  137.26  132.45  130.02  29.05  21.77  12.81  12.84 | R |
| **Chlorofyll a (Chl A)** | CH-5  CH-10  **CH-20** | 9.57  9.22  **8.41** | s  s  **s** | 137.6  108.2  93.4 | L |
| **Chlorofyll b (Chl B)** | **CHO-7**  CH-10 | **11.22**  9.72 | **s**  s | 178.2  109.4 | L |

In bold are evidenced the resonances chosen for metabolite quantification; s: singlet, d: doublet, t: triplet, q: quadruplet, dd: doublet of doublets, m: multiplet, bm: broad multiplet; L: leaves, R: roots

**Supplementary Table 3.** Phytochemical composition of chicory roots.

| Molecule | Amount (mg / 100 g) | | |
| --- | --- | --- | --- |
|  | MEB  Control | *Chaetomium globosum* 205 | *Minimedusa polyspora* 503 |
| Leucine | 0.19 ± 0.01 | 0.21 ± 0.03 | 0.23 ± 0.03 |
| Isoleucine | 0.4 ± 0.05 | 0.43 ± 0.08 | 0.45 ± 0.07 |
| Valine | 0.48 ± 0.06 | 0.64 ± 0.14 | 0.62 ± 0.09 |
| Threonine | 0.74 ± 0.11 | 1.03 ± 0.21 | 1.1 ± 0.25 |
| Alanine | 0.06 ± 0.05 | 0.36 ± 0.07 | 0.15 ± 0.09 |
| Glutamate | 5.64 ± 0.79 | 6.96 ± 0.93 | 7.21 ± 1.14 |
| Glutamine | 8.17 ± 1.64 | 12.33 ± 2.55 | 10.97 ± 2.20 |
| Pyroglutamate | 51.9 ± 6.69 | 58.38 ± 10.62 | 52.42 ± 8.45 |
| Asparagine | 1.46 ± 0.38 | 6.11 ± 3.42 | 3.14 ± 1.28 |
| GABA | 4.43 ± 0.56 | 4.17 ± 0.63 | 4.43 ± 0.5 |
| Tyrosine | 0.84 ± 0.44 | 0.93 ± 0.35 | 0.73 ± 0.17 |
| Phenylalanine | 0.51 ± 0.06 | 0.68 ± 0.06 | 0.63 ± 0.08 |
| Tryptophan | 0.36 ± 0.11 | 0.19 ± 0.07 | 0.11 ± 0.06 |
| 3-OHButyrate | 0.41 ± 0.03 | 0.54 ± 0.04 | 0.55 ± 0.05 |
| Acetate | 0.18 ± 0.04 | 0.16 ± 0.02 | 0.16 ± 0.02 |
| Malate | 2.93 ± 1.58 | 1.72 ± 0.49 | 1.76 ± 0.33 |
| Chicoric acid | 7.91 ± 3.18 | 16.74 ± 2.16 | 12.82 ± 2.62 |
| Chlorogenic acid | 2.33 ± 0.92 | 4.31 ± 0.40 | 3.59 ± 0.99 |
| Fumarate | 0.61 ± 0.20 | 0.51 ± 0.15 | 0.42 ± 0.16 |
| 4-OH-Benzoate | 0.26 ± 0.02 | 0.37 ± 0.06 | 0.4 ± 0.07 |
| Formate | 0.15 ± 0.02 | 0.12 ± 0.02 | 0.13 ± 0.02 |
| Glucose | 26.06 ± 3.47 | 25.26 ± 2.07 | 22.31 ± 4.84 |
| Sucrose | 221.65 ± 48.06 | 248.54 ± 45.89 | 226.44 ± 27.73 |
| Amylose | 325.09 ± 75.62 | 268.99 ± 49.84 | 336.92 ± 52.54 |
| β-Sitosterol | 3.39 ± 0.14 | 2.35 ± 0.12 | 2.64 ± 0.49 |
| Campesterol | 3.44 ± 0.24 | 2.9 ± 0.17 | 2.55 ± 0.29 |
| Oleic acid | 20.8 ± 2.34 | 20.82 ± 1.5 | 23.08 ± 4.87 |
| Linoleic acid | 18.29 ± 0.63 | 16.1 ± 0.93 | 15.7 ± 2.53 |
| Linolenic acid | 5.41 ± 0.21 | 3.99 ± 0.2 | 3.63 ± 0.54 |
| Triterpenes (eq. Oleanoic acid) | 3.97 ± 0.59 | 4.48 ± 3.3 | 0.9 ± 0.11 |
| Glycerol of monoacylglycerols | 0.24 ± 0.04 | 0.41 ± 0.05 | 0.37 ± 0.03 |
| Glycerol of phospholipids | 4.52 ± 0.52 | 2.14 ± 0.19 | 1.76 ± 0.49 |
| Choline | 2.81 ± 0.23 | 3.15 ± 0.36 | 3.4 ± 0.43 |
| Uracil | 1.35 ± 0.88 | 0.66 ± 0.14 | 0.86 ± 0.14 |
| Adenosine-phospate | 0.58 ± 0.09 | 0.64 ± 0.13 | 0.78 ± 0.19 |
| Trigonelline | 0.11 ± 0.03 | 0.08 ± 0.01 | 0.07 ± 0.01 |
| Xantophyl (eq. Luteolin) | 0.28 ± 0.02 | 0.23 ± 0.02 | 0.2 ± 0.03 |
| 7-OH-Coumarin | 0.39 ± 0.06 | 0.31 ± 0.08 | 0.34 ± 0.06 |
| Aldehyde (eq. Hexanal) | 0.1 ± 0.01 | 0.09 ± 0.01 | 0.09 ± 0.01 |

**Supplementary Table 4: Phytochemical composition of chicory leaves.**

| Molecule | Amount (mg / 100 g) | | |
| --- | --- | --- | --- |
|  | MEB  Control | *Chaetomium globosum* 205 | *Minimedusa polyspora* 503 |
| Leucine | 0.46 ± 0.03 | 0.32 ± 0.07 | 0.42 ± 0.04 |
| Isoleucine | 0.54 ± 0.05 | 0.34 ± 0.08 | 0.47 ± 0.03 |
| Valine | 0.7 ± 0.04 | 0.45 ± 0.06 | 0.64 ± 0.03 |
| Threonine | 0.84 ± 0.06 | 0.71 ± 0.04 | 0.85 ± 0.09 |
| Alanine | 1.18 ± 0.19 | 0.94 ± 0.11 | 1.01 ± 0.10 |
| Glutamate | 4.13 ± 0.99 | 3.77 ± 1.15 | 4.36 ± 0.96 |
| Asparagine | 3.74 ± 0.54 | 3.75 ± 0.35 | 4.67 ± 0.79 |
| GABA | 2.6 ± 1.42 | 1.84 ± 0.83 | 1.75 ± 0.65 |
| Lysine | 2.55 ± 0.26 | 2.46 ± 0.3 | 3.25 ± 0.76 |
| Tyrosine | 0.73 ± 0.18 | 0.93 ± 0.17 | 0.56 ± 0.22 |
| Phenylalanine | 0.54 ± 0.1 | 0.4 ± 0.09 | 0.7 ± 0.24 |
| Acetate | 0.25 ± 0.03 | 0.17 ± 0.04 | 0.13 ± 0.03 |
| Citrate | 181.88 ± 35.51 | 128.03 ± 13.23 | 140.6 ± 13.03 |
| Ascorbate | 6.30 ± 1.42 | 10.1 ± 2.49 | 8.61 ± 3.52 |
| Caffeic acid | 2.26 ± 0.63 | 3.12 ± 1.70 | 1.26 ± 0.64 |
| Chlorogenic acid | 45.62 ± 7.09 | 50.51 ± 10.78 | 46.07 ± 19.3 |
| Fumarate | 1.97 ± 0.38 | 1.37 ± 0.15 | 1.95 ± 0.21 |
| Chicoric acid | 11.56 ± 3.72 | 12.94 ± 5.98 | 12.42 ± 4.58 |
| 4-OH-Benzoate | 0.39 ± 0.04 | 1.27 ± 0.49 | 0.64 ± 0.09 |
| Formate | 0.23 ± 0.03 | 0.2 ± 0.02 | 0.17 ± 0.04 |
| Glucose | 13.09 ± 2.8 | 16.63 ± 4.41 | 13.57 ± 4.64 |
| Sucrose | 73.07 ± 7.63 | 112.66 ± 22.12 | 79.2 ± 11.77 |
| Amylose | 8.92 ± 3.05 | 24.64 ± 15.42 | 6.43 ± 1.33 |
| β-Sitosterol | 3.62 ± 0.23 | 2.7 ± 0.27 | 2.82 ± 0.19 |
| Campesterol | 1.91 ± 0.13 | 1.02 ± 0.16 | 0.93 ± 0.11 |
| Oleic acid | 26.16 ± 2.76 | 18.81 ± 1.38 | 23.25 ± 1.22 |
| Linoleic acid | 4.1 ± 0.59 | 4.11 ± 0.49 | 5.39 ± 0.66 |
| Linolenic acid | 64.91 ± 7.4 | 54.51 ± 4.54 | 66.56 ± 1.71 |
| Triterpenes (eq. Oleanoic acid) | 7.36 ± 4.08 | 0.75 ± 0.4 | 3.06 ± 0.79 |
| Glycerol of monoacylglicerol | 1.14 ± 0.48 | 2.49 ± 0.56 | 1.54 ± 0.39 |
| Glycerol of phospholipids | 2.01 ± 0.73 | 1.02 ± 0.46 | 2.01 ± 0.11 |
| Choline | 5.08 ± 0.5 | 4.78 ± 0.61 | 4.80 ± 0.20 |
| Uracil | 0.42 ± 0.19 | 0.17 ± 0.05 | 0.11 ± 0.07 |
| Adenosine-phospate | 0.64 ± 0.16 | 0.52 ± 0.09 | 0.72 ± 0.16 |
| Trigonelline | 0.5 ± 0.11 | 0.42 ± 0.06 | 0.35 ± 0.07 |
| Xantophyl (eq. Luteolin) | 3.58 ± 1.16 | 3.26 ± 0.34 | 4.15 ± 0.23 |
| Carotenoids | 3.12 ± 0.46 | 3.03 ± 0.29 | 3.53 ± 0.07 |
| Chlorophyll a | 40.98 ± 5.14 | 3.79 ± 0.67 | 7.07 ± 0.47 |
| Chlorophyll b | 9.96 ± 1.21 | 0.05 ± 0.02 | 5.18 ± 0.39 |

**Supplementary Figure 5.** PCA on the whole NMR data set obtained from leaf extracts

**Supplementary Figure 6.** PCA on the whole NMR data set obtained from root extracts
